# Cryo-EM structures of the eukaryotic replicative helicase bound to a translocation substrate

**DOI:** 10.1038/ncomms10708

**Published:** 2016-02-18

**Authors:** Ferdos Abid Ali, Ludovic Renault, Julian Gannon, Hailey L. Gahlon, Abhay Kotecha, Jin Chuan Zhou, David Rueda, Alessandro Costa

**Affiliations:** 1 Macromolecular Machines, Clare Hall Laboratory, The Francis Crick Institute, Blanche Lane, South Mimms EN6 3LD, UK; 2 National Institute for Biological Standards and Control, Microscopy and Imaging, Blanche Lane, South Mimms EN6 3QG, UK; 3 Section of Virology and Single Molecule Imaging Group, Department of Medicine, MRC Clinical Centre, Imperial College London, London W12 0NN, UK; 4 Division of Structural Biology, Wellcome Trust Centre for Human Genetics, University of Oxford, Roosevelt Drive, Oxford OX3 7BN, UK

## Abstract

The Cdc45-MCM-GINS (CMG) helicase unwinds DNA during the elongation step of eukaryotic genome duplication and this process depends on the MCM ATPase function. Whether CMG translocation occurs on single- or double-stranded DNA and how ATP hydrolysis drives DNA unwinding remain open questions. Here we use cryo-electron microscopy to describe two subnanometre resolution structures of the CMG helicase trapped on a DNA fork. In the predominant state, the ring-shaped C-terminal ATPase of MCM is compact and contacts single-stranded DNA, via a set of pre-sensor 1 hairpins that spiral around the translocation substrate. In the second state, the ATPase module is relaxed and apparently substrate free, while DNA intimately contacts the downstream amino-terminal tier of the MCM motor ring. These results, supported by single-molecule FRET measurements, lead us to suggest a replication fork unwinding mechanism whereby the N-terminal and AAA+ tiers of the MCM work in concert to translocate on single-stranded DNA.

## Abstract (web summary)

The Cdc45-MCM-GINS (CMG) helicase unwinds DNA during replication, a process that requires the ATPase-dependent activity of the MCM complex. Using cryo-EM reconstructions of the CMG complex in different conformations, the authors propose a model where the N-terminal and AAA+ domains of MCM work in concert to translocate along DNA.

## Graphical abstract

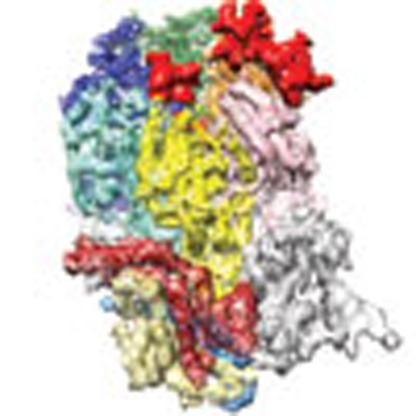

DNA replication onset requires an initiator that loads a set of two helicases for double-helix unwinding. This provides the single-stranded DNA template for the replicative polymerases. In eukaryotic cells, helicase recruitment and origin activation are temporally separated^1^. The origin recognition complex partakes in loading an inactive dimer of ring-shaped MCM helicase motors that encircle double-stranded DNA^2^,^3^,^4^. Origin firing depends on the recruitment of a set of replication factors^5^, including the GINS and Cdc45 activators that bind to each MCM ring in the dimer, forming a pair of multisubunit Cdc45-MCM-GINS (CMG) holo-helicases^6^,^7^. Upon origin activation, the two CMG particles are believed to separate and move in opposite directions to unwind DNA^8^, however, the molecular basis of MCM double-ring uncoupling is unknown. The MCM helicase motor is a ring-shaped hetero-hexamer containing six homologous polypeptides belonging to the superfamily of AAA+ ATPases. The N-terminal domain (NTD) of the MCM forms a DNA-binding collar and a co-axial carboxy-terminal ATPase motor powers substrate translocation through the ring central channel^9^. Whether DNA unwinding involves MCM motor translocation on duplex- or single-stranded DNA remains unclear^4^,^10^,^11^,^12^,^13^. DNA fork progression depends on the ATPase function of the MCM motor^5^,^14^, however, it is unknown how the energy derived from ATP hydrolysis is converted into motion and fork unwinding^15^. To start to address these outstanding questions, we have determined two cryo-electron microscopy (cryo-EM) structures of the CMG helicase trapped on a model DNA fork (by incubation with the slowly hydrolysable ATP analogue, ATPγS). We have also obtained two similar basic structures of the CMG helicase in the absence of DNA, imaged in conditions that allow for ATP turnover. Combined with single-molecule FRET analysis of DNA deformation by the CMG, our data provide important novel insights into the mechanism of replication fork progression in eukaryotic cells.

## Subnanometre resolution structure of the CMG

**Figure 1:**
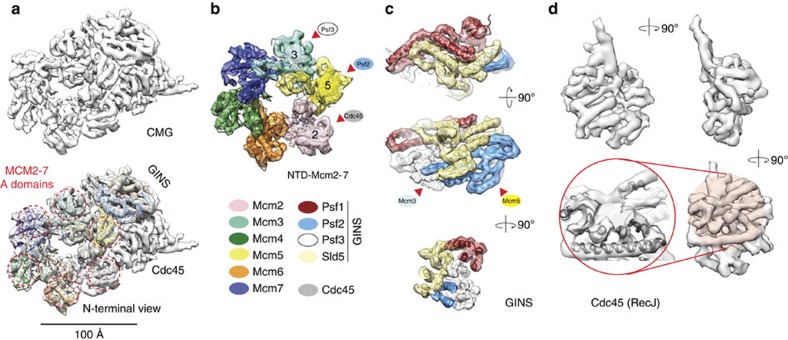
CMG helicase structure at subnanometre resolution. (**a**) Resolution density (7.4 Å) map of the CMG viewed from the MCM N-terminal face, without or with docked MCM and GINS atomic structures. (**b**) Detailed view of the MCM N-terminal DNA-interacting collar. Psf3 contacts Mcm3, Psf2 contacts Mcm5 and Cdc45 contacts Mcm2. (**c**) Detailed view of the GINS assembly with docked human atomic structure (PDB entry 2Q9Q). (**d**) Density assigned to Cdc45. The Cdc45 core matches the secondary structure elements of the bacterial RecJ exonuclease (PDB entry 1IR6).

Catalytically active, baculovirus-expressed *Drosophila melanogaster* CMG was incubated with a model replication fork in the presence of ATPγS, required for stable DNA binding^6^. Particles embedded in vitrified ice were imaged on a FEI Polara electron microscope equipped with an energy filter and a K2 Summit direct electron detector (Gatan, Inc.; Supplementary Fig. 1). Following two-dimensional (2D) and three-dimensional (3D) classification, a first structure was refined to 7.4 Å resolution (Supplementary Fig. 2). Atomic docking was employed to interpret the cryo-EM map, using the coordinates of known holo-helicase components. These efforts provide an exhaustive description of the CMG intersubunit interaction network. The structure contains a closed hexameric ring face that matches the N-terminal DNA-interacting collar of yeast MCM^4^ (Fig. 1a,b, PDB entry 3JA8), albeit with significant inter-domain rearrangements (Supplementary Fig. 3 and Supplementary Movie 1). Combined with previous subunit mapping studies^16^,^17^, our data confirm that GINS components Psf2 and Psf3 (PDB entry 2Q9Q) interact with the outer perimeter of MCM subunits 5 and 3 (Fig. 1a–c). Remarkably, Psf2 α-helices 3 and 5 (as defined in the human GINS structure^18^) contact a region of the Mcm5 N-terminal ‘A domain' that is protected by the N-terminal extension of the MCM subunit 7 from the opposing ring in the double hexamer, as described in the atomic resolution yeast structure^4^ (Supplementary Fig. 4).

**Figure 2:**
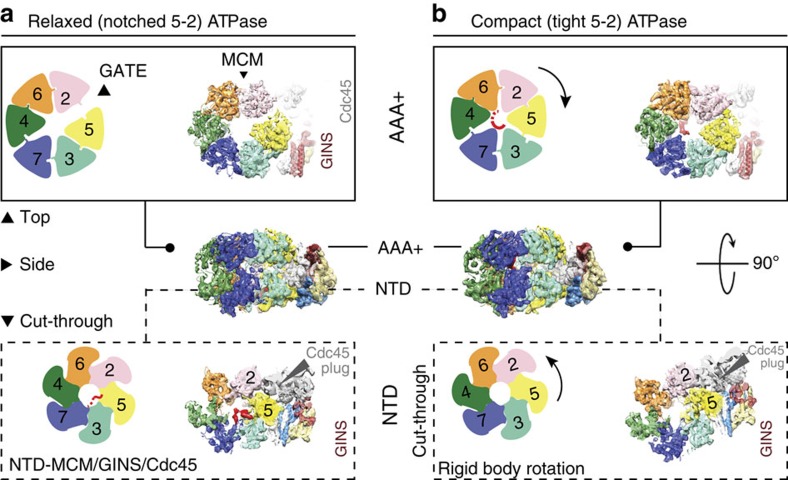
Two configurations in the ATPγS–CMG–DNA complex. (**a**) AAA+, side and cut-through view of the CMG in a relaxed ATPase configuration. Cdc45 topologically locks the Mcm5-2 gate. (**b**) AAA+, side and cut-through N-terminal view of the CMG in a compact ATPase configuration. DNA density surmounting the AAA+ domain has been removed for visualization purposes (also see Fig. 4).

As previously proposed^16^,^17^,^19^, unoccupied density mapping next to GINS is assigned to Cdc45 and indeed matches the secondary structure elements of RecJ^20^ (PDB entry 1IR6), a distant prokaryotic exonuclease homologue (Fig. 1d and Supplementary Fig. 5)^21^,^22^. In addition, a C-terminal protrusion projects from the RecJ-like catalytically defunct core of Cdc45 and wedges in between the Mcm5 and Mcm2 A domains^16^, as previously observed at low resolution^19^ (Figs 1a and 2a,b).

## AAA+ ATPase in the activated MCM helicase motor

DNA unwinding by hexameric helicases involves the ATP hydrolysis-driven reconfiguration of the ATPase centres, causing allosteric changes that promote substrate translocation^23^,^24^,^25^. Seeking to identify various intermediates in the DNA unwinding reaction, we refined all intact CMG 3D classes and identified two similar, higher-resolution CMG forms that differ only in their ATPase module. The first form has been introduced before, while a second form was refined to 9.8 Å resolution (Fig. 2a,b, Supplementary Figs 2,6 and 7, and Supplementary Movie 2). Inspection of the two helicase structures reveals that the ATPase tier is found as either topologically closed (‘compact ATPase') or notched (‘relaxed ATPase'). To define the interdomain arrangement in the helicase motor, we compared the recently established atomic model of yeast (double-hexameric) MCM^4^ with our newly determined CMG structures. The compact ATPase configuration is highly reminiscent of the yeast MCM, with a closed ATPase ring and nearly parallel NTD and AAA+ tiers. In this configuration, MCM protomers are aligned and NTD interacts with AAA+ in *cis*, with a slight left-handed twist^4^, confirming a previous model proposed by us^16^,^19^ and others^17^ (Fig. 2b). Conversely, in the relaxed ATPase form, the GINS-Cdc45 elements wedge in between the NTD and AAA+ tiers, a configuration previously observed at low resolution (Fig. 2a) (ref. 19). In this form, individual AAA+ domains become rearranged with respect to the cognate NTDs, approximating a conformer captured in a recent crystallographic study on an archaeal MCM hexamer^26^. DNA density can be recognized in both CMG structures and the nature of the DNA contacts will be discussed below.

## Remodelling of the ATPase motor

**Figure 3:**
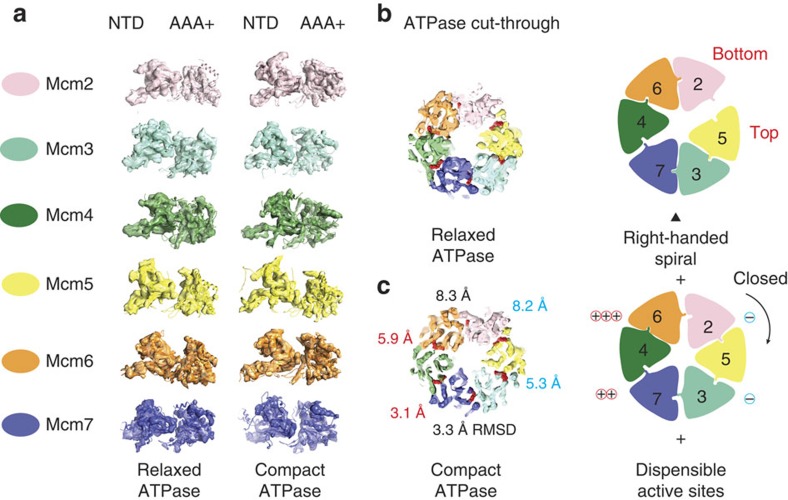
The MCM ATPase centres. (**a**) Segmented density and docked atomic structures of the six Mcm2-7 protomers in the two conformers. The NTD and AAA+ domains tilt and rock with respect to one another. (**b**) ATPase sites in the relaxed ATPase conformer. (**c**) ATPase sites in the compact ATPase conformer. One side of the MCM ring is more static and the corresponding active sites are dispensable for viability (−means Walker A mutation kills DNA unwinding, +means tolerated mutation).

To accurately describe ATPase motor dynamics, the yeast Mcm2-7 AAA+ hexamer was fitted into the CMG structure and further energy minimization was allowed for the individual docked ATPase modules into the two refined 3D classes. This docking exercise highlights a remarkable plasticity of the Mcm2-7 protomers, with relative tilting and rocking of the AAA+ domains, occurring both within the six Mcm2-7 ring subunits and between the two CMG conformers (Figs 2 and 3a). As previously proposed^16^,^19^, we can assign a discontinuity in the AAA+ domain to the Mcm5-2 ATPase interface (Fig. 2a,b). By comparing the compact and relaxed structures, a conformational transition can be modelled in the ATPase tier. This reveals two types of ATPase interfaces that behave differently. Movement mainly depends on the loosening and tightening of the Mcm5-2 interface (root mean squared deviation (r.m.s.d.) 8.2 Å between the open and closed configurations; Fig. 3b,c). Mcm5-2 loosening is chaperoned by compensatory tightening of the neighbouring Mcm3-5 and 2-6 interfaces (r.m.s.d.: 5.3 and 8.3 Å, respectively; Fig. 3b,c), while the rest of the subunits undergo more subtle reconfigurations (with Mcm4-7 and Mcm7-3 r.m.s.d.: 3.1 and 3.3 Å, respectively; Fig. 3b,c). Coherent with these asymmetric rearrangements, *in vitro* studies of the *Drosophila* CMG have shown that certain Mcm2-7 ATPase sites (found at the static hinge in our structures) can be inactivated with minimal effects on DNA unwinding by the CMG, while other sites (the active Mcm5-2 ATPase and its immediate neighbors) are required for translocation^6^ (Fig. 3c).

We have previously observed a compact Mcm5-2 interface at low resolution for a CMG in the presence of a non-hydrolysable ATP analogue (with or without DNA), while the nucleotide-free CMG contained a notched 5-2 interface^16^,^19^. These data support the notion that the relaxed ATPase form observed in our new, subnanometre resolution structure might contain an empty (post hydrolysis) ATPase site at the 5-2 interface. In line with this notion, the compact ATPase state is the predominant form (74% of particles that reach subnanometre resolution) in the ATPγS–CMG–DNA preparation, while the relaxed structure is underrepresented (the remaining 26%), as would be expected when using a slowly hydrolysable ATP analogue. To test whether ATP hydrolysis affects the particle distribution of the two conformers, we have solved the cryo-EM structure of the CMG incubated with ATP (hydrolysis rate is unaffected by the presence or absence of DNA^6^; imaging performed on a Titan Krios electron microscope, equipped with a Falcon II direct electron detector, FEI). Three-dimensional classification led to the identification of two CMG forms highly similar to the ATPγS/DNA-bound structures (although DNA free; Supplementary Figs 8–10 and Supplementary Movies 2 and 3). Remarkably, when the ATP-treated CMG is imaged, the notched Mcm5-2 structure is more represented than the compact state (72% versus 28%), supporting the notion that ATP hydrolysis by the Mcm5-2 site could convert the compact ATPase into a relaxed configuration (Supplementary Fig. 11). Although we cannot rule out the possibility that one of the two forms might be an inactive state of the helicase^12^, it is possible that the two structures described here represent two intermediates of the DNA translocation reaction.

## The CMG helicase is a single-stranded DNA translocase

**Figure 4:**
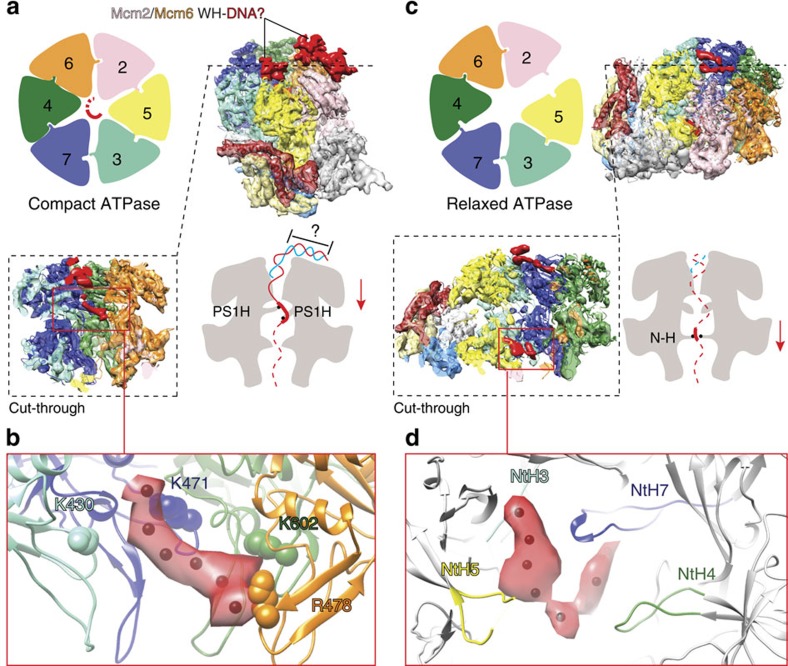
DNA-bound form of the CMG helicase. (**a**) The compact ATPase form contains rod-shaped, bent density features surmounting the ATPase face tentatively assigned to duplex DNA engaged by the MCM winged helix (WH) C-terminal extensions. An MCM slice through the side view reveals an extended density feature, which we assign to single-stranded DNA, traversing the AAA+ pore. (**b**) Single-stranded DNA contacts conserved positively charged residues on the AAA+ PS1 hairpins that have a key role in DNA unwinding. (**c**) The relaxed ATPase form contains a thin density feature surmounting the AAA+ ring, which we assign to the flexible C-terminal MCM WH extensions. Although the AAA+ tier appears substrate free, a well-resolved elongated density threads through the MCM N-terminal collar and (**d**) contacts a set of N-terminal hairpins important for DNA binding and helicase activity.

**Figure 5:**
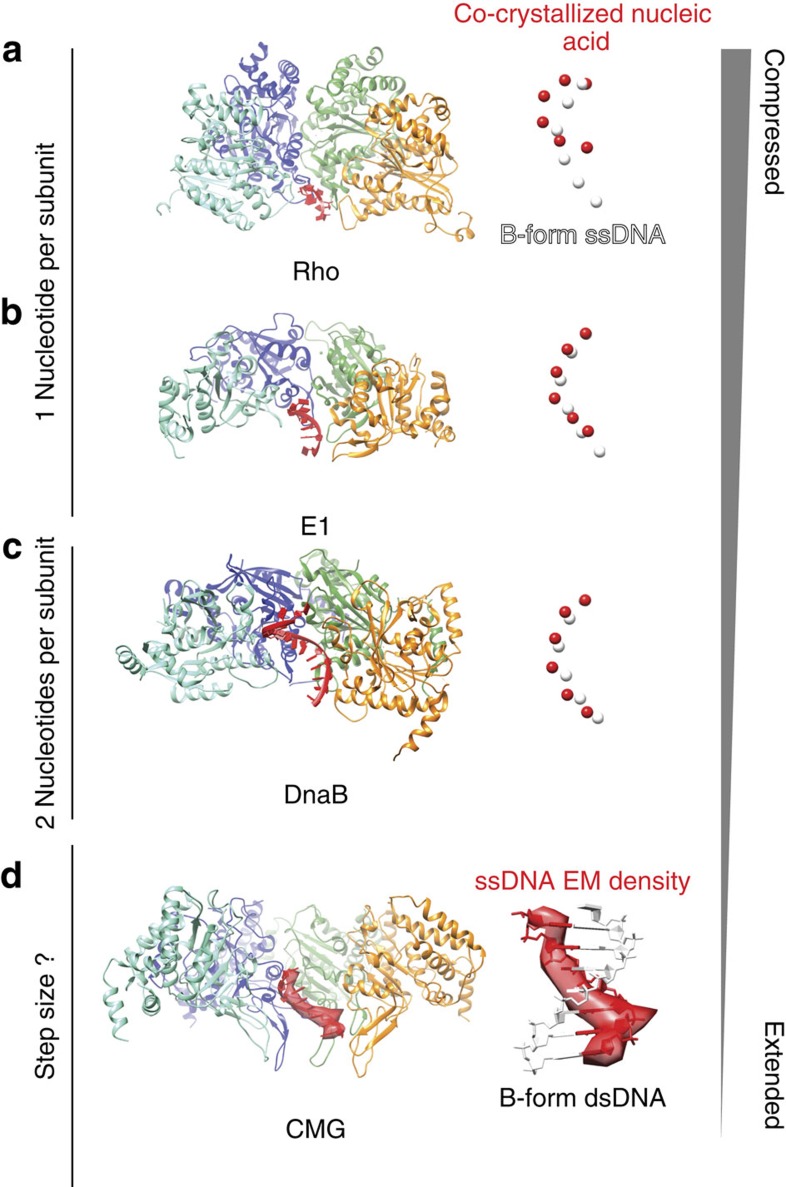
Comparison of the ATPγS–CMG–DNA structure with available helicase–nucleic acid assemblies. (**a**) The Rho termination factor (PDB entry 3ICE) contacts and compresses single-stranded RNA. The RNA structure is compared with one single DNA strand extracted from a B-form double helix. Spheres represent the centre of mass of each nucleotide. (**b**) The Papillomavirus E1 replicative helicase (PDB entry 2GXA) contacts and compresses single-stranded DNA. (**c**) The bacterial DnaB replicative helicase contacts and slightly compresses single-stranded DNA (approximating A-form DNA, PDB entry 4ESV). (**d**) The MCM motor of the CMG harbours a more extended form of single-stranded DNA, which appears to match B-form DNA. The DNA density matches the structure of one single strand in the double helix. This supports the notion that the CMG is a single-stranded DNA translocase.

Comparison of the compact ATPase DNA–CMG structure with the DNA-free counterpart reveals the presence of apparently bent, rod-shaped density features contacting the C-terminal face of Mcm2-6 and surmounting the pore of the MCM motor (Fig. 4a and Supplementary Fig. 11). The local resolution is low in this region of the cryo-EM map (compared with the core protein components) and not suitable for atomic docking (Supplementary Figs 6 and 7). Nonetheless, the dimensions and overall shape of the additional density feature matches that of duplex DNA, possibly engaged by one or more C-terminal MCM extensions (containing a winged helix fold)^4^. The notion that duplex DNA enters through the AAA+, and not the N-terminal tier of MCM, is in agreement with our earlier streptavidin labelling of the DNA fork^19^. The inner core of the AAA+ domain is better defined and shows continuous density spanning three MCM subunits (Mcm7-4-6) and contacting the pre-sensor 1 (PS1) and helix-2 insert pore loops (Fig. 4b). This density can accommodate single- but not double-stranded DNA (Fig. 5), indicating that the AAA+ domain of the CMG helicase is a single-stranded DNA translocase (Supplementary Movie 3). A set of positively charged residues on the PS1 hairpins (Mcm7 K471, Mcm4 K602 and Mcm6 R478) contact and spiral around the nucleic acid (Fig. 4b). These DNA-binding elements are highly conserved from archaea through eukarya and their mutation affects DNA binding and abrogate DNA unwinding^11^,^12^,^27^. Noticeably, Mcm3 K430 is also poised in close proximity to the DNA density (Fig. 4b). This is a known DNA translocation element that is essential for viability in yeast^27^.

Comparison of the relaxed ATPase structures in the presence or absence of DNA shows more subtle differences (Supplementary Fig. 11). In both structures, thin density features surmount the C-terminal face of the AAA+ pore and probably correspond to DNA-free winged helix MCM appendices^4^, raising the question of whether DNA occupancy in this state is lower than in the compact ATPase state. Remarkably, although the AAA+ pore loops appear DNA free, a second set of N-terminal pore loops encircle an elongated density feature visible in the nucleic-acid-treated CMG but not in the DNA-free CMG. Thus, we assign the N-terminal contacting density to DNA. Similar to the PS1 hairpins, these ‘N-terminal hairpins' are also conserved and are essential for DNA binding and have a role in unwinding^28^,^29^,^30^ (Fig. 4d). It remains to be established whether the compact and relaxed DNA-binding modes in the ring channel correspond to a translocation and a paused state^12^, respectively, or represent two intermediates in the DNA translocation reaction.

**Figure 6:**
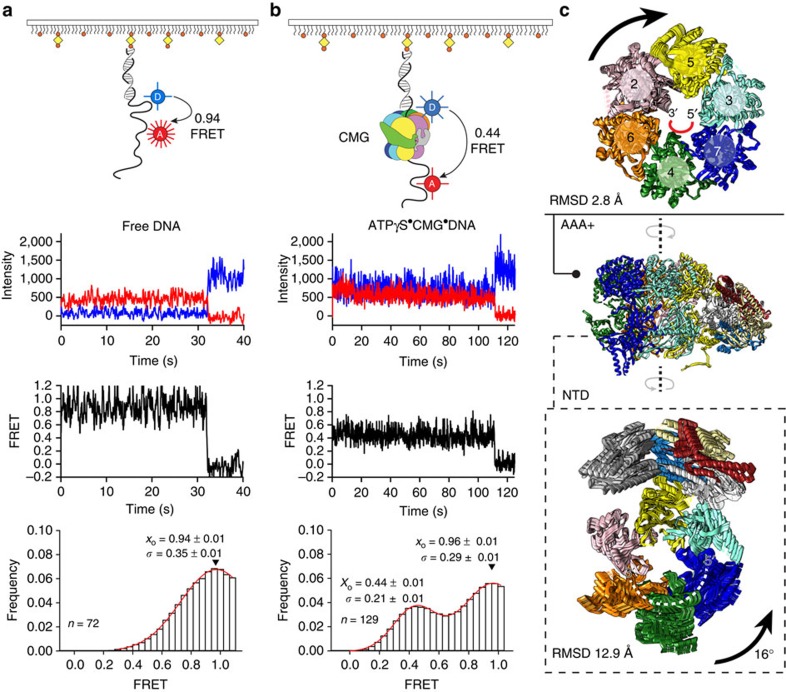
DNA engagement and deformation by the CMG helicase. Single-molecule FRET analysis of (**a**) isolated DNA and (**b**) DNA+ATPγS CMG. Top to bottom: cartoon schematic of the single-molecule FRET experiment indicating FRET between Cy3 (Donor, blue sphere) and Cy5 (Acceptor, red sphere, 7-nt separation) attached to biotinylated 3′-tailed duplex DNA, immobilized on a neutravidin-coated biotin-PEG surface; donor (blue) and acceptor (red) intensity trajectories are anti-correlated until single-step photobleaching of the acceptor. FRET trajectories (black) between Cy3 and Cy5 exhibit a sharp drop to zero FRET when the acceptor photobleaches; histogram of FRET values collected from all molecules with a Gaussian fit are shown in red, where *x*_0_ is the mean FRET and σ is the distribution width. ATPase controlled ring rotation and DNA stretching by the CMG helicase. (**c**) The AAA+ tier of the Mcm2-7 motor rotates clockwise as Mcm2 moves towards the Mcm5 protomer, to close a gap in the motor domain. The DNA-interacting NTD rotates anticlockwise as a rigid body, together with GINS and Cdc45.

Comparison of the DNA-engaged MCM ATPase tier with other structures of nucleic acid-bound hexameric helicases highlights a mixture of similarities and differences (Fig. 5). A universal feature appears to be the presence of a right-handed spiral formed by the ATPase pore loops, which follows the helical character of the nucleic acid substrate^15^. Whereas in previously reported helicase structures (Rho, E1 and, to a lesser extent, DnaB) the translocation substrate is more compressed than that of canonical B-form DNA^24^,^31^,^32^ (Fig. 5a–c), the ATPγS–CMG–DNA complex matches the structure of one single strand extracted from a B-form double helix (Fig. 5d). Noticeably, the Rho and E1 helicases, which significantly compress the translocation substrate as they encircle it, contact one base per ATPase protomer, while DnaB contacts two bases per protomer. These observations are compatible with an unwinding step size of 1- (Rho and E1) and 2-bp (DnaB) per ATP hydrolysed. Although the resolution of our compact ATPγS–CMG–DNA complex is not high enough to count bases in the single-stranded nucleic acid, the more extended state of the DNA substrate suggests that the CMG might unwind at least two base pairs per ATP molecule hydrolysed. To confirm the notion that single-stranded DNA is stabilized in an extended configuration on CMG binding, we performed single-molecule experiments, in the presence of either ATPγS or ATP. CMG–DNA binding was monitored by measuring FRET efficiency between a Cy3 donor and a Cy5 acceptor fluorophore, separated by seven nucleotides in surface-immobilized single-stranded DNA. This is an established method to measure DNA stretching by multi-subunit, DNA-encircling ATPases^33^ (Fig. 6a,b). CMG binding to DNA in the presence of ATPγS results in a dramatic decrease from 0.94±0.01 (naked DNA; Fig. 6a) to 0.44±0.01 (DNA+CMG; Fig. 6b and Supplementary Fig. 12) in mean FRET (*x*_0_), due to both a reduction in molecular flexibility relative to naked single-stranded DNA and CMG-mediated single-stranded DNA stretching. Similar results were obtained with ATP (*x*_0_=0.96±0.01 for naked DNA and *x*_0_=0.48±0.01 for CMG), although binding/stretching events occurred with lower frequency (Supplementary Fig. 12). These data agree with the observation that use of a slowly hydrolysable ATP analogue is required to stabilize the interaction between the MCM AAA+ domain and single-stranded DNA^6^,^12^, and support the notion that CMG binding stabilizes and stretches single-stranded DNA. We note that the lower frequency of DNA binding/stretching observed when CMG is ATP incubated correlates with the predominance of the relaxed ATPase form observed in the ATP–CMG cryo-EM data set.

## ATPase controlled rotation of the CMG DNA-binding domain

How does the ATPase state of the MCM motor affect the rest of the CMG structure? To address this question we have aligned the AAA+ rings of the two ATP–CMG conformers and used molecular morphing to model a transition from a relaxed to a compact ATPase conformation. In our model, the motor undergoes minor subunit rearrangements, with an overall r.m.s.d. of 2.8 Å, mainly due to the breathing of the Mcm5-2 interface (Fig. 6c). Conversely, the non-catalytic portion of the CMG complex, formed by Cdc45, NTD-Mcm2-7 and GINS, rotates by 16° as a rigid body with respect to the motor (overall r.m.s.d. of 12.9 Å between the two forms; Figs 2a,b and 6c, and Supplementary Movie 4). Remarkably, the NTD and AAA+ tiers of Mcm2-7 rotate in opposite directions, as they transition from a relaxed to a compact ATPase form (Fig. 6c). This rotation is compatible with a nucleotide-state-controlled inter-subunit movement, previously observed using double electron–electron resonance in the archaeal MCM motor^29^.

Previous crystallographic studies in archaea have shown that single-stranded DNA can line the NTD-MCM pore. This interaction (preserved in the eukaryotic Mcm6-4-7) is essential for DNA replication initiation in yeast and probably important for the initial melting of the origin DNA duplex (Fig. 6c). According to our model, as the ATPase motor tightens to engage the translocation strand, the AAA+ domain rotates clockwise, while the NTD DNA-interacting collar of the MCM rotates anticlockwise. Considering that DNA interacts with the inner perimeter of NTD-MCM running 3′→5′ in an anticlockwise manner^30^, we note that a further anticlockwise NTD rotation would result in DNA duplex underwinding, indeed compatible with origin DNA opening.

## Discussion

**Figure 7:**
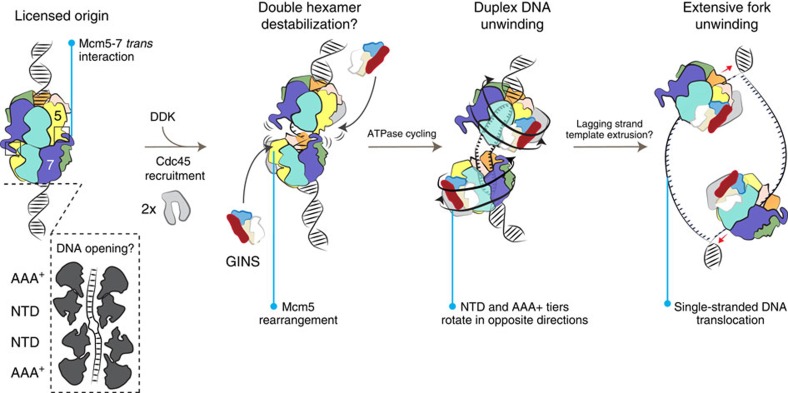
Origin activation and replication fork unwinding by the CMG helicase. Origin licensing involves the loading of a head-to-head double hexameric ring that encircles duplex DNA, which might become partially deformed. Cdc45 is loaded onto the double hexamer, in a process that requires DDK phosphorylation of MCM. DDK phosphorylation might cause a rearrangement in Mcm5 and disrupt the (yeast specific) Mcm5-7 *trans* interaction. This rearrangement would expose a GINS interacting element in Mcm5. ATP hydrolysis by the MCM promotes the relative rotation of the NTD and AAA+ tiers of the helicase, in a movement that might promote duplex DNA underwinding. Following a poorly understood lagging-strand extrusion process, the CMG helicase extensively unwinds the replication fork, by translocating on single-stranded DNA.

DNA replication start sites need to be licensed for initiation and this process involves the deposition of a pair of MCM helicases that form a two-fold symmetric, double-hexameric assembly^2^,^3^. GINS/Cdc45 recruitment is necessary for origin firing and multiple lines of evidence suggest that two CMG holo-helicases separate and move in opposite directions during replication. For example, single-molecule studies have demonstrated that two replisomes need not be physically linked for efficient DNA replication to occur^8^. Further evidence of helicase separation is derived from EM studies on the CMG, which primarily form monomeric assemblies^16^ (although loosely tethered dimeric CMGs have also been observed)^19^. A recent high-resolution study describes the dimerization interface of the yeast MCM double ring, which is in part formed by an N-terminal Mcm7 helical insert that latches onto the Mcm5 A domain in the opposing hexamer^4^. Our subnanometre resolution CMG structure reveals that the A domain of Mcm5 undergoes a reconfiguration in the CMG (Supplementary Movie 1), which would be incompatible with Mcm7 engagement (at least in yeast). Remarkably, the highly conserved Mcm5 site that is generally protected by Mcm7 in the double hexamer is found GINS associated in the CMG (Supplementary Fig. 4). GINS recruitment depends on the DDK phosphorylation of Mcm4, 6 and 7 (refs 5, 34); however, DDK requirement in yeast can be bypassed by a mutation of Mcm5 (ref. 35) that promotes a reconfiguration of the Mcm5 A domain^28^. DDK phosphorylation probably promotes a similar conformational change in Mcm5 that could disrupt the (yeast specific) Mcm5-7 double hexamer interface and enable GINS binding. These events, we suggest, might help destabilize the double hexamer and contribute to replication intiation (Fig. 7).

Our new CMG structures better explain the role of Cdc45, containing a globular extension emanating from the RecJ-like core. This extension inserts in between the A domains of Mcm5 and 2 (refs 16, 19, 36), plugging a gate that is generally found open in the isolated MCM complex^16^,^36^ and used for DNA loading^37^. During elongation, ring opening is thought to allow for the bypass of a roadblock on the translocation strand in certain viral replicative helicases^38^, while the eukaryotic replisome stalls on meeting an obstruction on the translocation strand^10^,^39^. A topologically sealed Mcm2-7 ring (the CMG structure described here) provides a rationale to explain this observation. With Cdc45 plugging the Mcm5-2 gate, full ring opening is energetically disfavoured in the holo-helicase (Fig. 2a,b), possibly explaining helicase stalling.

Whether the CMG helicase is a single-stranded or a duplex DNA translocase is still debated^4^,^9^,^10^,^40^. As the Mcm2-7 is initially loaded onto duplex DNA, replication fork establishment has been suggested to occur via two alternative mechanisms. According to the ‘strand extrusion' model duplex, DNA enters the AAA+ ATPase tier of MCM and the two strands become spatially segregated before exiting the helicase toroid^9^,^13^. A second model, called ‘strand exclusion', envisages translocation on one single strand and steric exclusion of the other strand^9^,^10^,^11^ (Supplementary Fig. 13). Recent structural characterization of the yeast MCM double hexamer provides some evidence in support of the strand extrusion model^4^. In the double-ring structure, a unique lateral channel exists between the Mcm6 and 2 subunits, which might be used for extrusion of the lagging strand. Given the position of this side pore, mapping in between the AAA+ and NTD tiers, strand extrusion would occur after the double strand has been spooled through the AAA+ motor (Supplementary Fig. 13). This hypothesis is supported by the notion that the CMG helicase contains a central pore that is large enough to accommodate double-stranded DNA^16^,^17^,^19^. Our new ATPγS–CMG–DNA structure, however, provides robust evidence against a duplex translocase model and favours the steric exclusion mechanism. In fact, in the compact ATPase state we observe thin continuous density that spirals inside the AAA+ MCM hexamer and contacts a set of positively charged residues known to be important for DNA binding and translocation. This density can accommodate single- but not double-stranded DNA (Fig. 5d), indicating that the MCM motor unwinds a replication fork by translocating on the leading strand template, whereas the lagging strand template is sterically excluded (Fig. 7). Support for our model derives from studies on *Xenopus* egg extracts, according to which the replisome can bypass a road block on the lagging strand but not on the translocation (leading) strand^10^. Future, high-resolution studies on higher-order assemblies will be needed to define the replication fork trajectory in the eukaryotic replisome^17^.

Three main mechanisms have been proposed for coupling nucleotide turnover with substrate translocation in hexameric NTPases, involving either stochastic, concerted or rotary firing^15^. The stochastic model, mainly supported by studies on protein unfoldases, suggests that the NTPase firing does not need to follow an ordered sequence of events for substrate translocation to occur^41^,^42^. The concerted model envisions six NTPase subunits that simultaneously bind, hydrolyse or release the nucleotide. This mechanism is supported by symmetrized crystallographic structures of the SV40 large T-antigen replicative helicase^23^. According to the rotary mechanism, nucleotide hydrolysis occurs sequentially from one hydrolase centre to the next, similar to a Mexican wave around a stadium^15^,^24^,^31^. The rotary cycling model is supported by biochemical and crystallographic studies of the T7 gp4 helicase^43^ and by three helicase structures, the Rho transcription termination^24^ factor, the Papillomavirus E1 (ref. 31) and the bacterial DnaB replicative helicases^32^, which have all been imaged bound to their translocation substrate (single-stranded nucleic acid). Importantly, in the E1 and Rho translocation–substrate complexes, the hydrolase centres are radially distributed in an order that sequentially visits the steps of the NTPase cycle^24^,^31^. In both structures, a staircase formed by the ATPase pore loops follows the right-handed spiral of a single-stranded nucleic acid and the position of the pore loops in the assembly correlates with the nucleotide occupancy state of the ATPase centres. An ATPase-controlled rotary pore-loop movement would therefore promote substrate translocation through the ATPase channel. Owing to the homo-oligomeric nature of the E1 helicase complex however, a rotary cycling movement in the helicase hexamer can only be modelled and not visually proven. Because of its inherently asymmetric nature, we reasoned that the Mcm2-7 hetero-hexamer would be a useful tool to test the rotary cycling model. The asymmetric distribution of ATPase centres and the ordered spiral organization of the DNA-interacting PS1 hairpins indeed favour a sequential rotary over a concerted firing or stochastic mechanism for substrate translocation by the CMG. However, to our surprise, we were unable to characterize multiple rotational states of the ATPase, with DNA contacting different ATPase protomers. Rather, the ATPase exists in two states: compact (DNA gripping) and relaxed (DNA ungripping; Supplementary Fig. 14). Our results lend themselves to two possible explanations. A first possibility is that one of six rotational states of the MCM is more stable than the other, short-lived ATPase ring permutations. However, we favour a second possibility. The ATPase motor could fluctuate between the compact state (where DNA is stabilized/extended by Mcm7-4-6) and the relaxed state where the AAA+ motor releases the substrate. In this second configuration, DNA could be handed off to the N-terminal collar and our observation of stable NTD–DNA association in the relaxed ATPase structure supports this model. Alternatively, the relaxed ATPase configuration could represent a stalled form of the helicase, as could be found, for example, at a pausing replication fork (Supplementary Fig. 14).

Notably, not all ATPase sites in the MCM motor of the CMG equally contribute to DNA translocation^6^,^44^ and this feature is shared by other hetero-hexameric AAA+ ATPases such as the Rpt1-6 assembly^45^ of the proteasome or the dynein motor (which in some organisms contains ATPase centres that have become inactivated during evolution)^46^. Both the proteasome and dynein have been imaged in a compact and a relaxed ATPase form, with movement dependent on the breathing of one lone hydrolase interface and chaperoning activity of the neighbouring interfaces^47^,^48^ (such as the Mcm5-2 site and its immediate neighbours in our CMG structure). Although these ATPase motors are functionally very distinct (a DNA, a polypeptide and a microtubule translocase, respectively), phylogenetic^49^,^50^ and mechanochemical^19^ kinship has been noticed before for the three systems, suggesting a shared mechanism of substrate translocation for hetero-hexameric AAA+ ATPases. Taken together, our results suggest that the CMG helicase might translocate on single-stranded DNA via a mechanism distinct from strictly sequential rotary cycling^6^,^44^ and probably involves the interplay between the N-terminal and AAA+ tiers of the MCM motor^29^.

## Methods

### Purification of the CMG helicase

pFastBac1 plasmids containing genes that encode the individual subunits of the CMG complex (a gift from Dr Michael R. Botchan) were used to produce bacmids, which were subsequently transfected into Sf9 cells using the Invitrogen Bac-to-Bac Baculovirus Expression System methods^6^. P3 baculoviruses used in protein expression experiments were freshly amplified from the P2 stocks for 5 days in 100 ml of Sf9 cells grown in 250 ml flasks in Graces medium supplemented with 10% FCS. The infections for protein purification were carried out by inoculating 4 l of Hi5 cells at 10^6^ ml^−1^ with a multiplicity of infection of 5 by adding 200 ml of each virus stock at a titre 10^8^ pfu ml^−1^. Infected cells incubated for 72 h at 27 °C were harvested by centrifugation and washed with PBS+5 mM MgCl_2_. The following steps were performed either on ice or at 4 °C, unless indicated otherwise. The collected cells were resuspended in 200 ml of buffer C (25 mM Hepes pH 7.6, 0.02% Tween-20, 10% glycerol, 1 mM EDTA, 1 mM EGTA) supplemented with 15 mM KCl, 2 mM MgCl_2_, 0.4 mM phenylmethylsulfonyl fluoride, 2 mM 2-mercaptoethanol and the complete protease inhibitors cocktail from Roche Diagnostics. The cell suspension was snap frozen in 10 ml aliquots and stored at −80 °C. To purify CMG complexes from the extract, the infected cell suspension was thawed and cells were broken in a Dounce homogenizer. KCl was added to 100 mM and the extract was cleared by centrifugation at 14,000 r.p.m. in an Avanti J-26S XP centrifuge for 10 min. The cleared extract was incubated with 2 ml of anti-FLAG M2-agarose beads (Sigma-Aldrich) for 2–3 h with continuous end-over-end mixing. The beads were then collected in a 20-ml Poly-Prep disposable chromatography column (BioRad) and washed in 30 ml C-100 buffer (buffer C with 100 mM KCl and 1 mM dithiothreitol (DTT)) before transferring to a 10 ml column (BioRad). The column was washed twice with 5 ml C-100 buffer. Bound complexes were eluted with 200 μg ml^−1^ flag peptide in C-100 supplemented with complete protease inhibitor cocktail, the first elution step was performed in 5 ml of elution buffer for 15 min at room temperature with end-over-end mixing, then collecting the flowthrough and repeating the step with 4 ml of elution buffer for 10 min. Both fractions were pooled, cooled on ice and pumped through a Mono S HiTrap SP FF column equilibrated in C-100 buffer. The flowthrough and a further 4 ml of wash was collected and injected onto a MonoQ HR 5/5 column equilibrated in C-100 buffer^6^. The column was washed in 15 ml of C-100 and bound complexes were eluted with 20-ml 100–550 mM KCl gradient in buffer C supplemented with 1 mM DTT. Fractions (0.5 ml) were collected, the CMG peak fractions (eluted at 410–440 mM KCl) were diluted to 150 mM KCl in buffer C and injected onto a Mono Q PC 1.6/5 column connected to AKTAmicro purification system equilibrated in buffer D (25 mM Hepes pH 7.6, 1 mM EDTA, 1 mM EGTA, 1 mM DTT) supplemented with 150 mM KCl. Elution was performed using a 2-ml 150–550 mM KCl linear gradient in buffer D and 75 μl fractions were collected. The CMG peak fractions were pooled and dialysed for 16 h into buffer A (25 mM Hepes pH 7.6, 50 mM sodium acetate, 10 mM magnesium acetate, 1 mM DTT). The CMG preparation was supplemented with 1 mM ATP or treated as described in the ‘CMG–DNA complex reconstitution' section. Protein concentration was measured by using known MCM3 protein standards serving as a reference on a silver stain SDS–PAGE gel. A yield of 150 μl with a concentration of 1 μM was achieved.

### Helicase assay

Assays were performed in 20 μl reactions contacting 25 mM Hepes pH 7.5, 75 mM NaCl, 0.5 mM ATP, 10 mM magnesium acetate, 1 mM DTT, 0.1 mg ml^−1^ BSA. Purified CMG (123 to 410 fmol) was incubated in the presence of 27 μM ATPγS and 1.8 pmol of [γ-^32^P]-ATP-labelled fork DNA substrate for 3 h at 30 °C, to allow for fork loading. Unwinding was induced by adding ATP to 7 mM for 5 min at 30 °C and stopped with 6 × stop buffer (150 mM Tris pH 8.0, 3% SDS, 120 mM EDTA). The unwinding product was separated using 8% PAGE in 0.1% SDS 1 × TBE.

### CMG–DNA complex reconstitution

The oligonucleotides used for CMG–DNA reconstitution were synthesized by Integrated DNA Technology. Sequences of the two partially complementary oligos used are shown below:

Leading strand template: 5′-CACTCGGGCTCGTTTTACAACGTCGTGACTGGGCACTTGATCGGCCAACCTTTTTTTTTTTTTTTTTTTTTTTTTTTTTTTTT TTTTTTT-3′

Lagging strand template: 5′-CTGGCGTCGGGTCGGCGGTTGGCCGAT CAAGTGCCCAGTCACGACGTTGTAA AACGAGCCCGAGTG-3′

When annealed together, this model replication fork contains a 50-bp double-stranded region with two single-stranded fork overhangs (16-nt on the 5′-end and 40-nt poly-T on the 3′-end, based on Petojevic *et al.*^12^).

Annealing of the two strands was performed by mixing each of the oligos in equimolar amounts, heating at 95 °C for 3 min and slow cooling to room temperature for 60 min. To reconstitute the nucleoprotein complex, forked DNA substrate was added to dialysed ∼300 nM CMG in a 2:1 molar ratio in the presence of 0.1 mM ATPγS (Sigma). The CMG–DNA–ATPγS mixture was left to incubate for 2 h at 30 °C, to allow for complex association before cryo-grid preparation.

### Cryo-grid preparation and data collection for CMG–DNA

Four microlitres of reconstituted CMG–DNA assembly at 300 nM concentration were applied onto freshly glow-discharged Quantifoil 1.2/1.3 or C-flat 1/1 grids. After a 30-s incubation in 100% humidity, the Quantifoil grid was double-side blotted for 4 s using a Vitrobot (FEI) and plunged into liquid ethane. For the C-flat grid, the sample was incubated for 2 min and double-side blotted for 3 s in a Cp3 (Gatan, Inc.), operating at 90% humidity and plunged into liquid ethane. Cryo-grids were screened for ice quality using on a JEOL-2100 or a FEI Spirit LaB6 operated at 120 kV and equipped with an 4 k × 4 k or 2 k × 2 k Ultrascan charge-coupled device camera (Gatan, Inc.), respectively. Data were collected on a Tecnai F30 Polara electron microscope operated at 300 kV and equipped with a K2 Summit direct electron detector (Gatan, Inc.) and an energy filter in zero-loss mode (GIF Quantum, Gatan, Inc.). Twenty-five-frame movies (2,098) were manually collected, with a single frame duration of 0.4 ms. Movies were acquired using SerialEM, in single-electron counting mode with a total dose of 48 e^−^ Å^−2^ and a −3.5 to 1.7 defocus range, at a constant nominal magnification of × 37,037, yielding a 1.35-Å pixel size.

### Cryo-grid preparation and data collection for ATP–CMG

The ATP–CMG sample at 330 nM concentration was applied onto freshly glow-discharged open-holes Quantifoil 1.2/1.3. After 30 s incubation, grids were double-side blotted for 5 s in a Vitrobot (FEI) at 100% humidity and plunge frozen into liquefied ethane. Grids were loaded onto the LMB Cambridge Titan Krios electron microscope (FEI) operated at 300 kV for automated data collection with the EPU software (FEI). Images were recorded on a FEI Falcon II detector at a nominal magnification of × 47,000 (yielding a pixel size of 1.77 Å). An in-house built system described in ref. 51 was used to collect 17 frames per second. Five hundred and thirty-six movies of 51 frames were recorded using a −2 to −4 μm defocus range with an electron dose of 51 e^−^ Å^−2^.

### CMG–DNA image processing

To correct for beam-induced drift, whole frame alignment and averaging was performed for each movie using MotionCorr (http://cryoem.ucsf.edu/software/driftcorr.html)^52^. Particles (340,573) were picked semi-automatically in EMAN2 (ref. 53). Contrast transfer function parameters were estimated using CTFFIND4 (ref. 54) and low-quality integrated movies were excluded. All further processing was performed in RELION 1.4 (ref. 55). Extracted particles were binned by 2, yielding a pixel size of 2.7 Å per pixel. Two-dimensional classification allowed for the isolation of a set of 60,287 high-quality CMG particles (Supplementary Fig. 2). A first 3D refinement was performed using a 50-Å low-pass filtered compact ATP–CMG structure as a starting model, resulting in an initial 8.1 Å structure. Postprocessing (using automatically estimated B factor and an arbitrarily chosen mask) improved the resolution to 7.2 Å but resulted in a poorly defined ATPase tier (Supplementary Fig. 2). To separate various ATPase conformers in the data set, the refined 3D volume was filtered to 50 Å and used as a model for 3D classification without particle alignment (ten classes). After excluding poor-quality classes, 2 structures were isolated, referred to as ‘compact ATPase' (38,792 particles; Supplementary Fig. 6) and ‘relaxed ATPase' (13,692 particles; Supplementary Fig. 7, also refer to Figs 2 and 4, and Supplementary Fig. 2 for comparisons). Each particle subset was separately refined and postprocessed, resulting in a 7.4-Å resolution structure (compact ATPase) and 9.8 Å (relaxed ATPase).

### ATP–CMG image processing

Similar to the CMG–DNA data set, beam-induced drift was corrected and averaging was performed for each movie as described in ref. 52. Contrast transfer function parameters were estimated using CTFFIND3 (ref. 56) and the best 471 out of 536 integrated movies were selected for particle picking in XMIPP3 (ref. 57) and subsequent processing in RELION 1.3 (ref. 55). Semi-manually picked particles (160,401) were 2D-classified, to get rid of bad particles, resulting in a clean data set containing 78,601 particles. A first ATP–CMG 3D volume was determined to 9.5 Å, using a 50-Å-filtered version of EMDB entry 2,772 as an initial model (Supplementary Fig. 8). Using the 9.5-Å structure filtered to 50 Å, 3D classification was performed to identify four recognizable CMG classes^58^ as the new, ‘3D-cleaned' data set. Using statistical movie processing^59^, an 8.3-Å reconstruction (Supplementary Fig. 8) was then obtained from these merged (29,772) particles. As the resulting structure showed a disordered AAA+ motor domain, further focused 3D classification^55^,^58^ was performed to identify different motor conformers. This resulted in two classes (compact ATPase, 5,111 particles and relaxed ATPase, 13,182 particles), which were independently refined and postprocessed as described above (Supplementary Figs 8–10).

### Resolution estimation and model building

Resolution of the obtained cryo-EM maps was estimated using the ‘gold-standard' Fourier Shell Correlation (FSC) method, using the 0.143 FSC criterion. Local resolution was estimated using ResMap^60^ and visualized using UCSF Chimera^61^. Automated atomic docking was performed using UCSF Chimera and further model manipulation performed using The PyMOL Molecular Graphics System. The A and B–C domains of the NTD tier of the yeast Mcm2-7 structure (PDB entry 3JA8 (ref. 4)) were docked as isolated rigid bodies. GINS (PDB entry 2Q9Q^18^) and RecJ (PDB entry 1IR6 (ref. 20)) were docked as rigid bodies. The C-terminal Psf1 domain GINS was modelled as described in ref. 19. The AAA+–MCM domains were docked as six individual rigid bodies after docking of the cryo-EM MCM AAA+ tier (PDB entry 3JA8 (ref. 4)). FSC to compare the CMG atomic models and the cryo-EM maps were computed using RELION 1.4 and visualized with the PDBe FSC server. Figures and Movies were generated using UCSF Chimera^61^.

### Single-molecule FRET

DNA oligonucleotides were purchased from Operon and labelled with fluorescent dyes Cy3 and Cy5. Reactions were carried out using an amino-modified C6-dT oligonucleotide and a mono-reactive Cy3 or Cy5 dye (GE Healthcare). Labelling reactions were performed with 1 nmol of DNA in 44 μl of 100 mM sodium carbonate buffer pH 8.5 and 10 nmol of Cy3 or Cy5 dye dissolved in 7 μl of dimethyl sulfoxide. Reactions were performed overnight at room temperature. Reverse-phase HPLC purification was performed on an analytical C8-column (Sigma-Aldrich Supelco Discovery BIO wide pore C8, 25 cm × 4.6 mm × 5 μm), to separate labelled and unlabelled DNA; fractions containing labelled DNA were collected and stored at −20 °C in 10 mM Tris-HCl pH 8.0. DNA sequences are as follows; for the Cy3 strand 5′-Cy3-CGCGAGGAATGGATGTAGGG-biotin-3′ and for the Cy5 strand 5′-CCCTACATCCATTCCTCGCGTTTTTT (Cy5-T)(T)_65_-3′. Quartz slides and cover slips were prepared following established protocols^62^. Briefly, quartz slides and coverslips were passivated with methoxy-PEG-SVA (*M*_r_=5,000, Laysan Bio, Inc.) containing 10% biotin-PEG-SVA (*M*_r_=3,400, Laysan Bio, Inc.) in 100 mM sodium bicarbonate. Reaction chambers were first incubated with 0.2 mg ml^−1^ BSA (Sigma-Aldrich) in T50 buffer (10 mM Tris-HCl pH 7.0 and 50 mM NaCl) for 10 min. Next, BSA was washed with T50 buffer and neutravidin (0.2 mg ml^−1^ in T50 buffer) was injected and incubated for 10 min. Excess neutravidin was removed by washing with buffer E (25 mM Hepes pH 7.6, 50 mM sodium acetate, 10 mM magnesium acetate, 10% glycerol, 1 mM DTT and 2 mM Trolox). DNA was surface-immobilized by incubation for 10 min with an annealed biotinylated DNA duplex (25 pM). Excess DNA was then washed with imaging buffer containing the CMG protein (Buffer E supplemented with 50 μM ATPγS (Sigma-Aldrich) or ATP (Sigma-Aldrich), 7 nM CMG protein and an oxygen scavenging system containing 10 mM 3,4-dihydroxybenzoic acid (Sigma-Aldrich) and 120 nM protocatechuate dioxygenase (Sigma-Aldrich; the final concentration of protocatechuate dioxygenase is corrected for the presence of 40% stabilizer), to minimize dye photobleaching). DNA molecules were imaged on a home-built, prism-based total internal reflection fluorescence microscope. All single-molecule measurements were recorded at room temperature using continuous green excitation (532 nM laser) at ∼1.0 mW and 30 ms time resolution. Apparent FRET efficiencies were calculated as FRET=*I*_A_/(*I*_D_+*I*_A_), where *I*_A_ and *I*_D_ are the acceptor and donor intensities, respectively. Acceptor intensity (*I*_A_) was corrected for donor emission in the acceptor channel (11%), no direct excitation of the acceptor was observed.

## Additional information

**Accession codes:** ATP–CMG: compact ATPase EMDB entry EMD-3320, relaxed ATPase EMDB entry EMD-3321. ATPγS–CMG–DNA: compact ATPase EMDB entry EMD-3318, relaxed ATPase EMDB entry EMD-3319.

**How to cite this article:** Abid Ali, F. *et al.* Cryo-EM structures of the eukaryotic replicative helicase bound to a translocation substrate. *Nat. Commun.* 7:10708 doi: 10.1038/ncomms10708 (2016).

## Supplementary Material

Supplementary Figures Supplementary Figures 1-14

Supplementary Movie 1 N-terminal view of the DNA interacting collar as it transitions from the Mcm2-7 to the CMG configuration.

Supplementary Movie 2 Cross sections through the relaxed and compact ATP-CMG and ATPγS-CMG-DNA maps.

Supplementary Movie 3 Cut-through views of the relaxed and compact ATP-CMG and ATPγS-CMG-DNA structures. Structures are viewed from the AAA+ (top) to the N-terminal domain (bottom).

Supplementary Movie 4 Interpolation between the two relaxed and compact ATP-CMG states.
