# Supplementary material for: Cryo-EM structures of the eukaryotic replicative helicase bound to a translocation substrate
Source: Nat Commun. 2016 Feb 18;7:10708. doi: 10.1038/ncomms10708 (PMC4759635; doi:10.1038/ncomms10708)
Supplement: Supplementary Figures — 1-14 [file ncomms10708-s1.pdf]

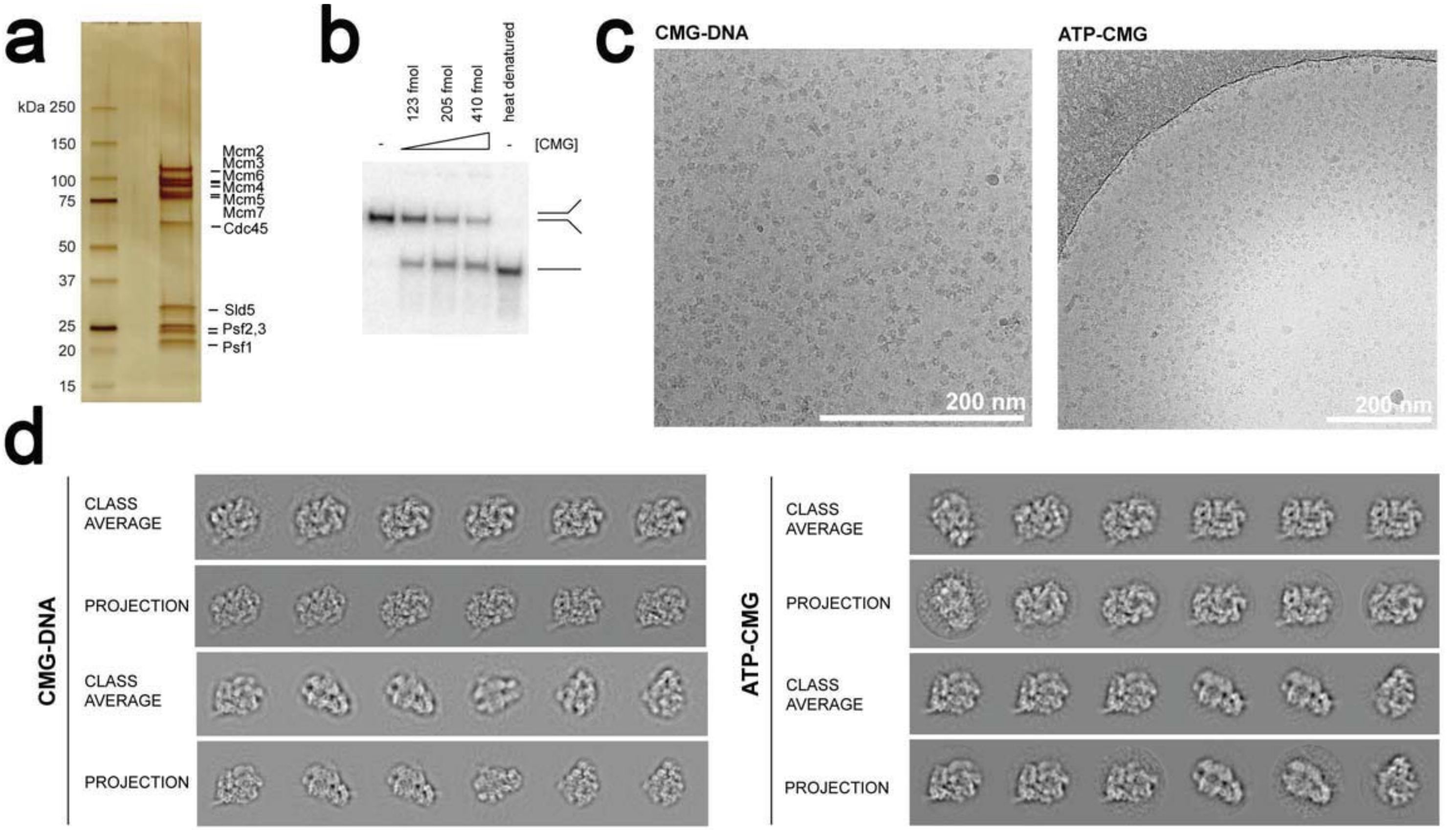

Supplementary Figure-1 (Costa)

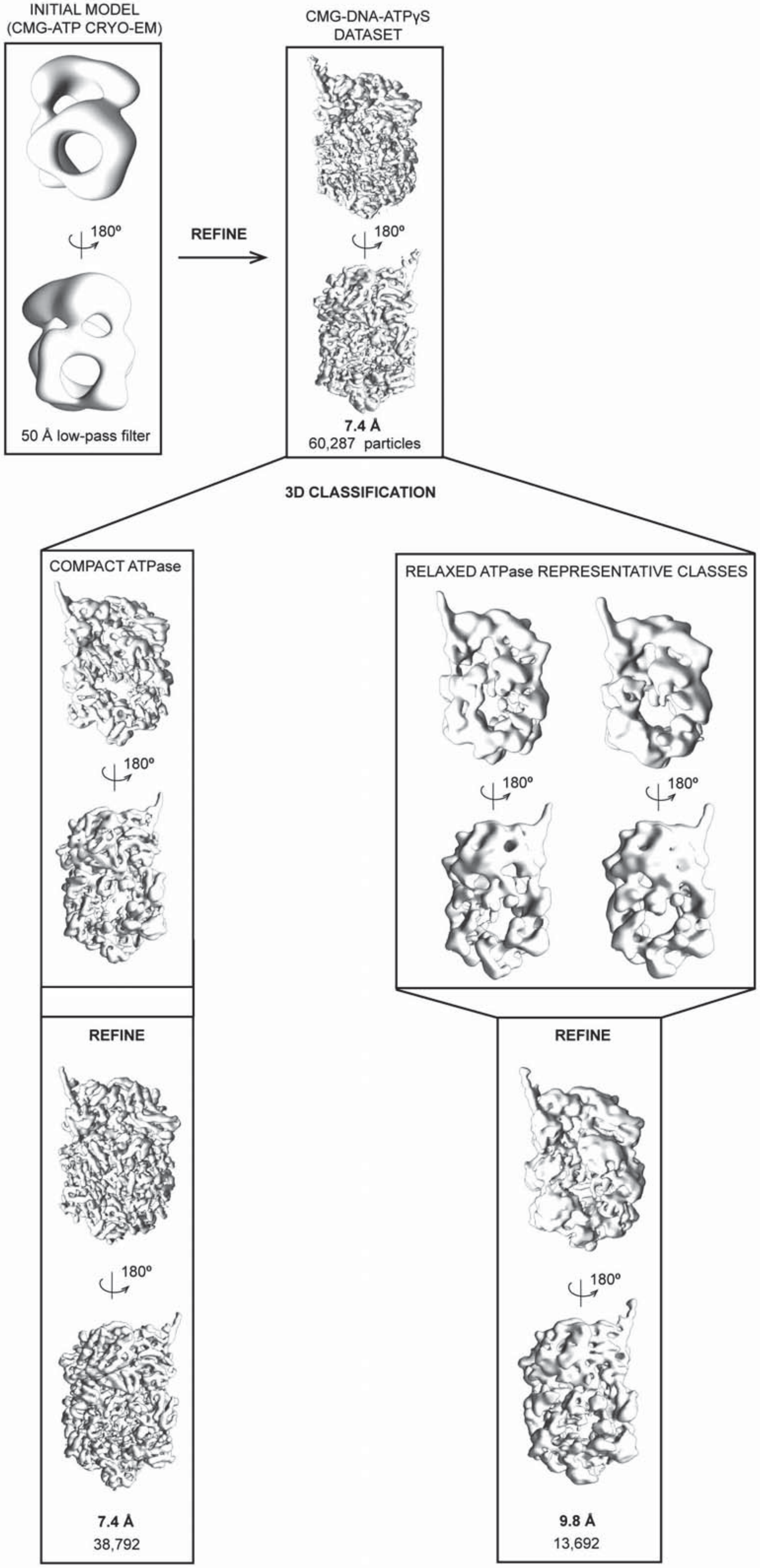

Supplementary Figure-2 (Costa)

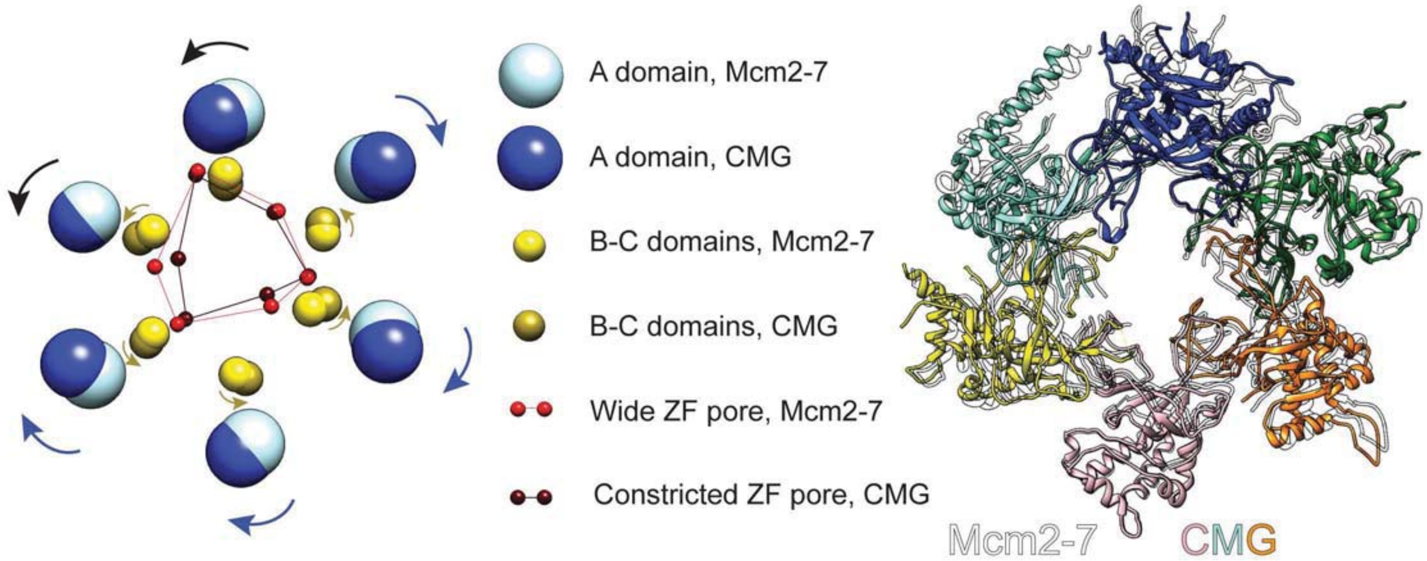

Supplementary Figure-3 (Costa)

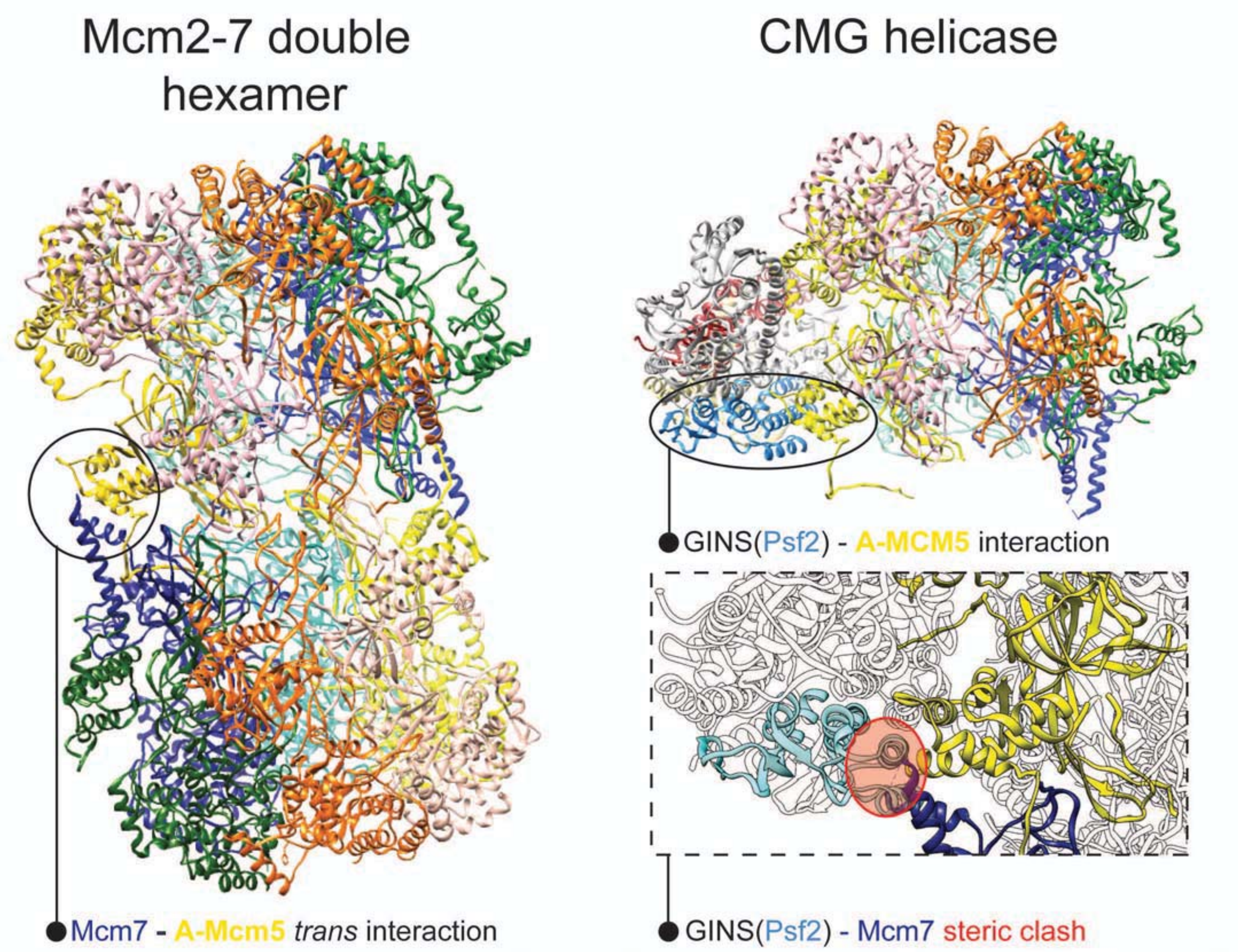

Supplementary Figure-4 (Costa)

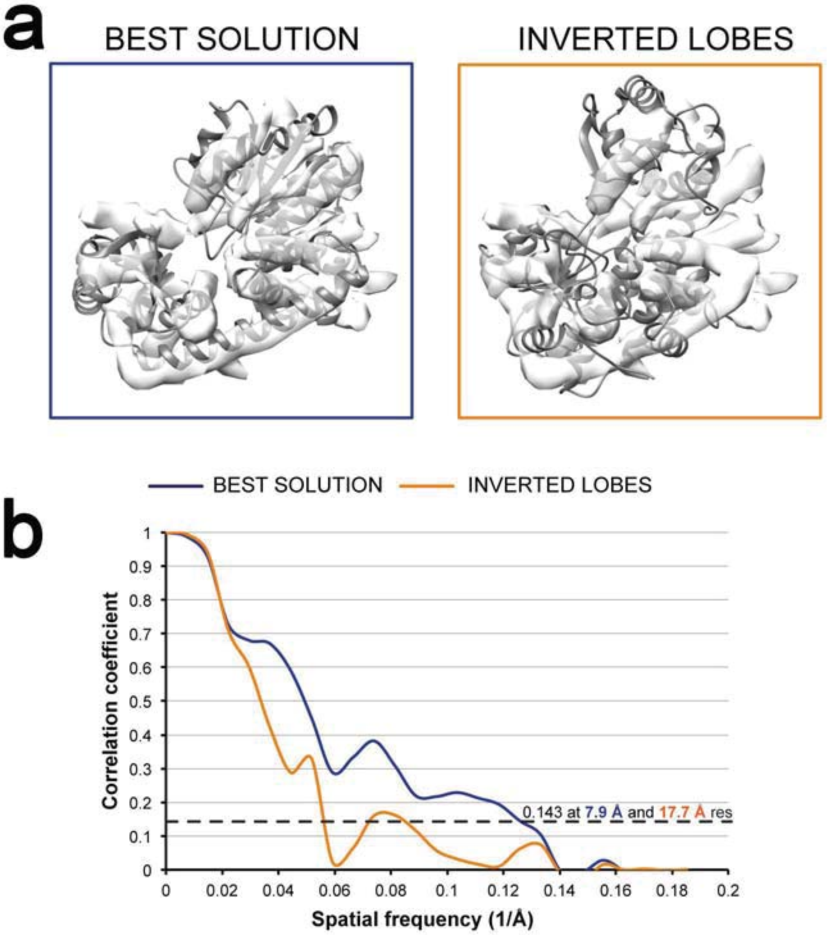

Supplementary Figure-5 (Costa)

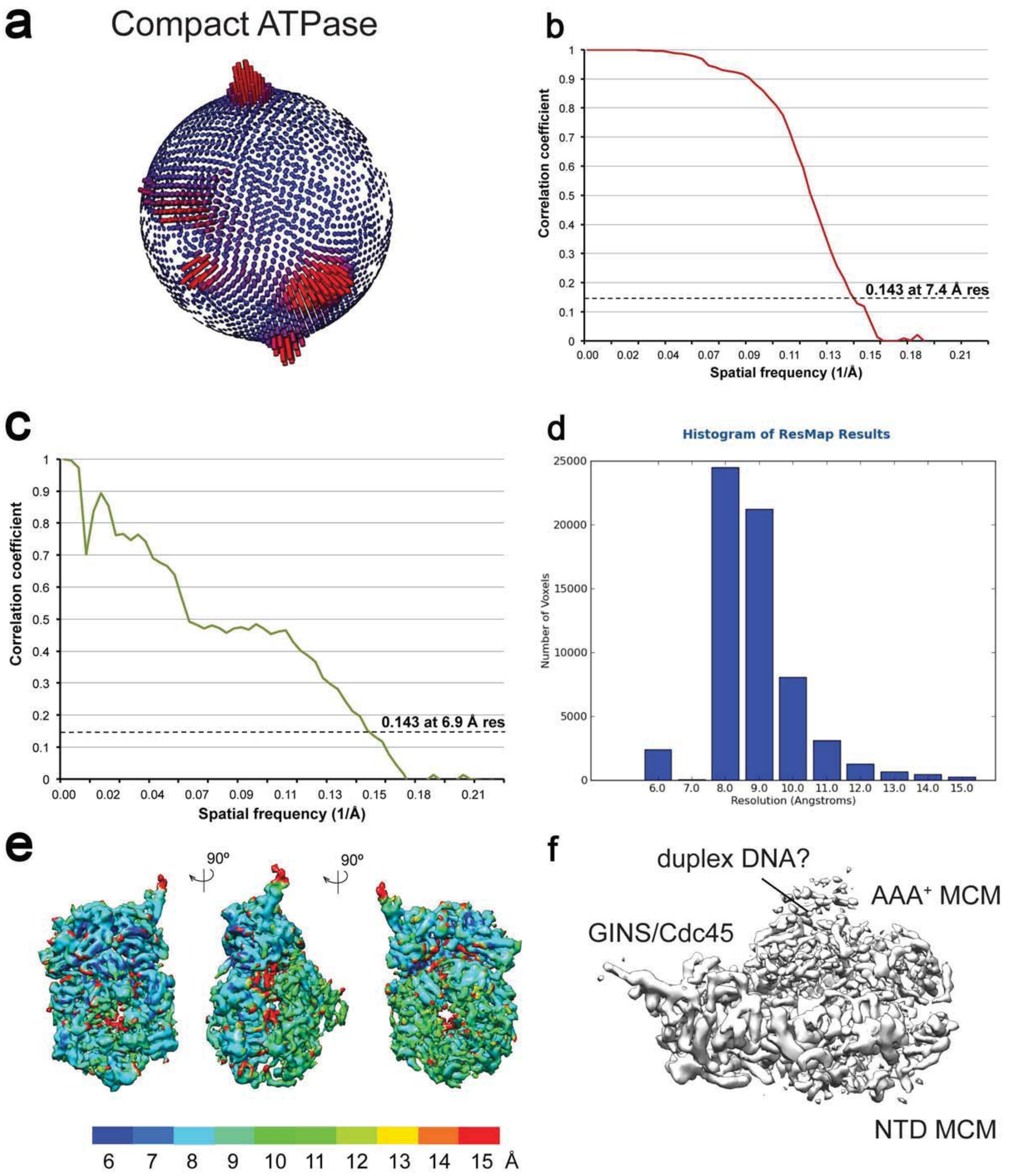

Supplementary Figure-6 (Costa)

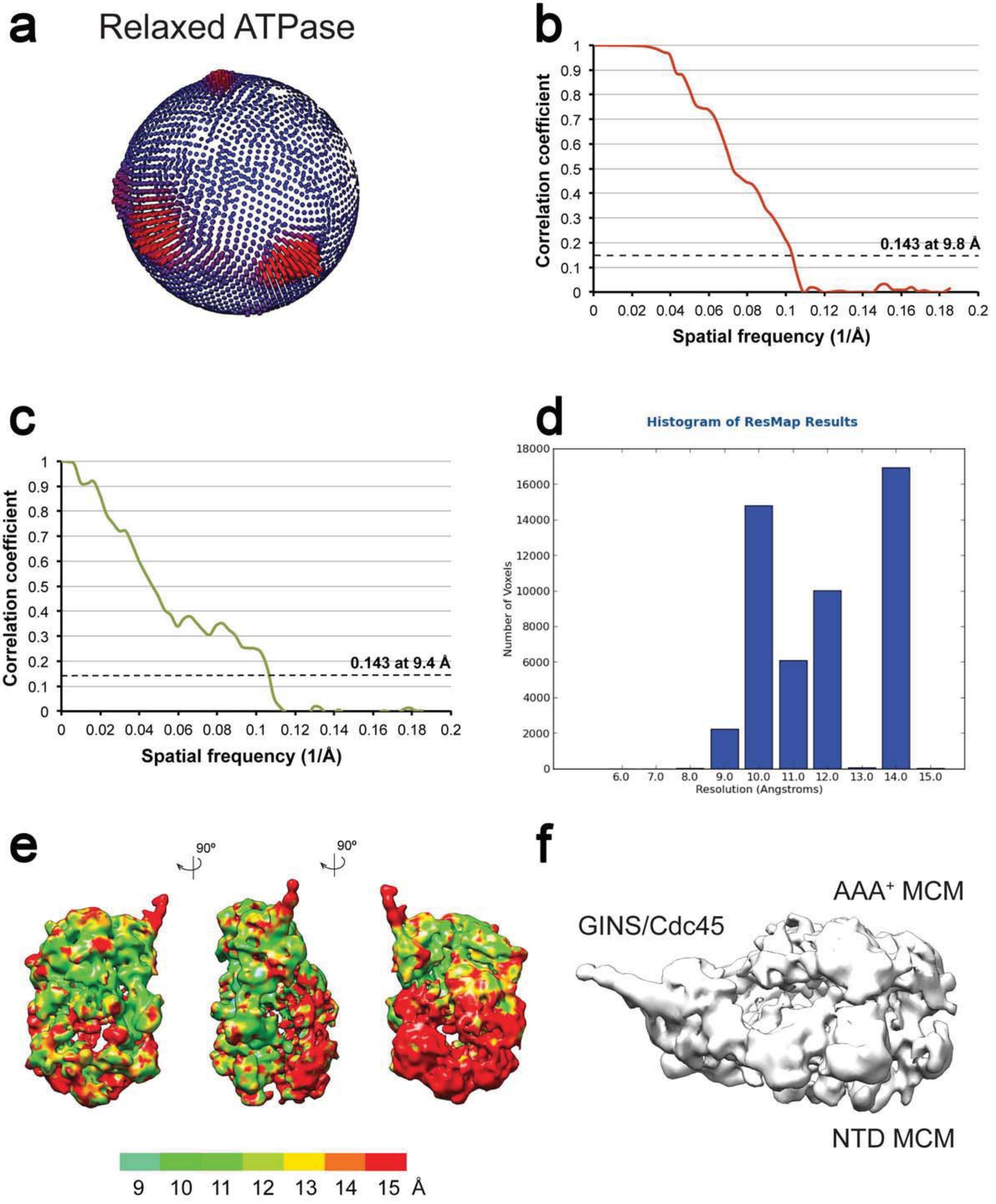

Supplementary Figure-7 (Costa)

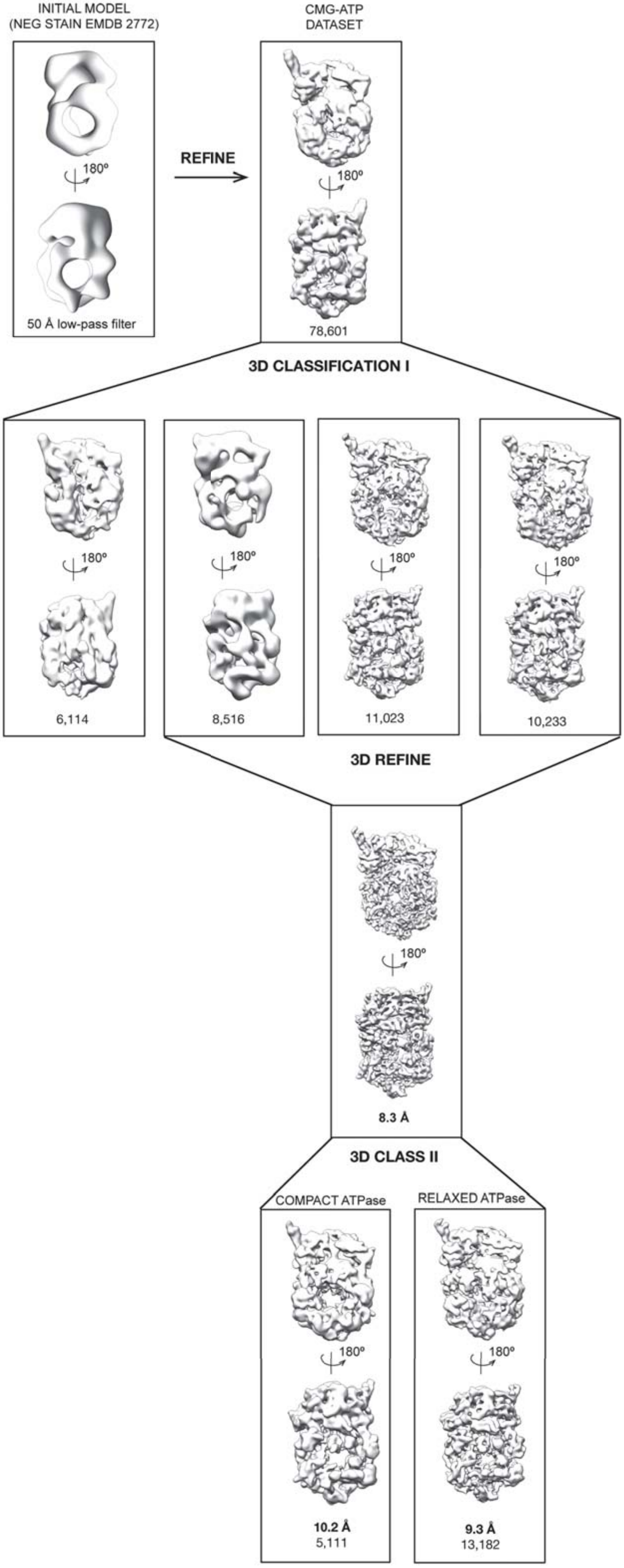

Supplementary Figure-8 (Costa)

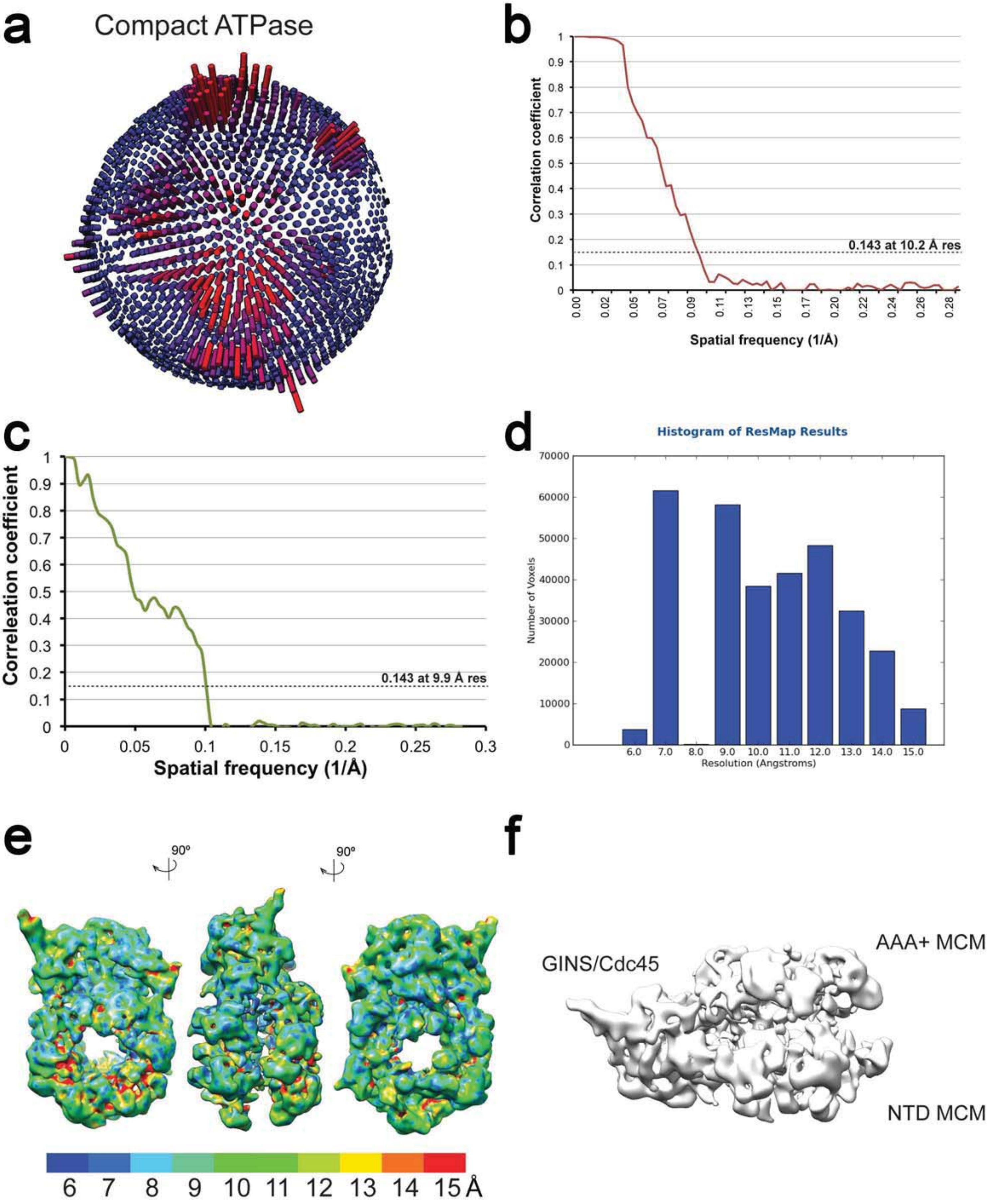

Supplementary Figure-9 (Costa)

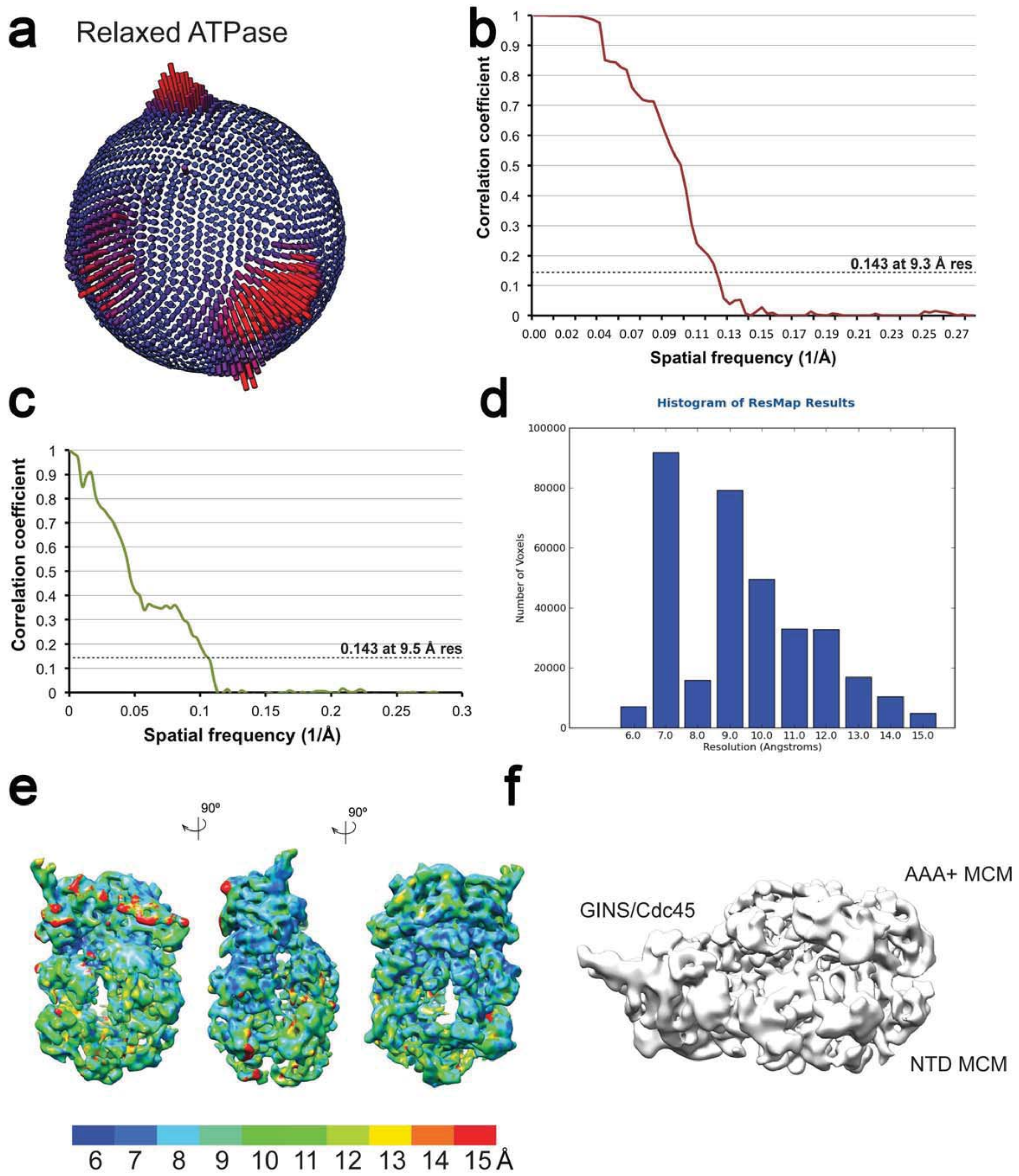

Supplementary Figure-10 (Costa)

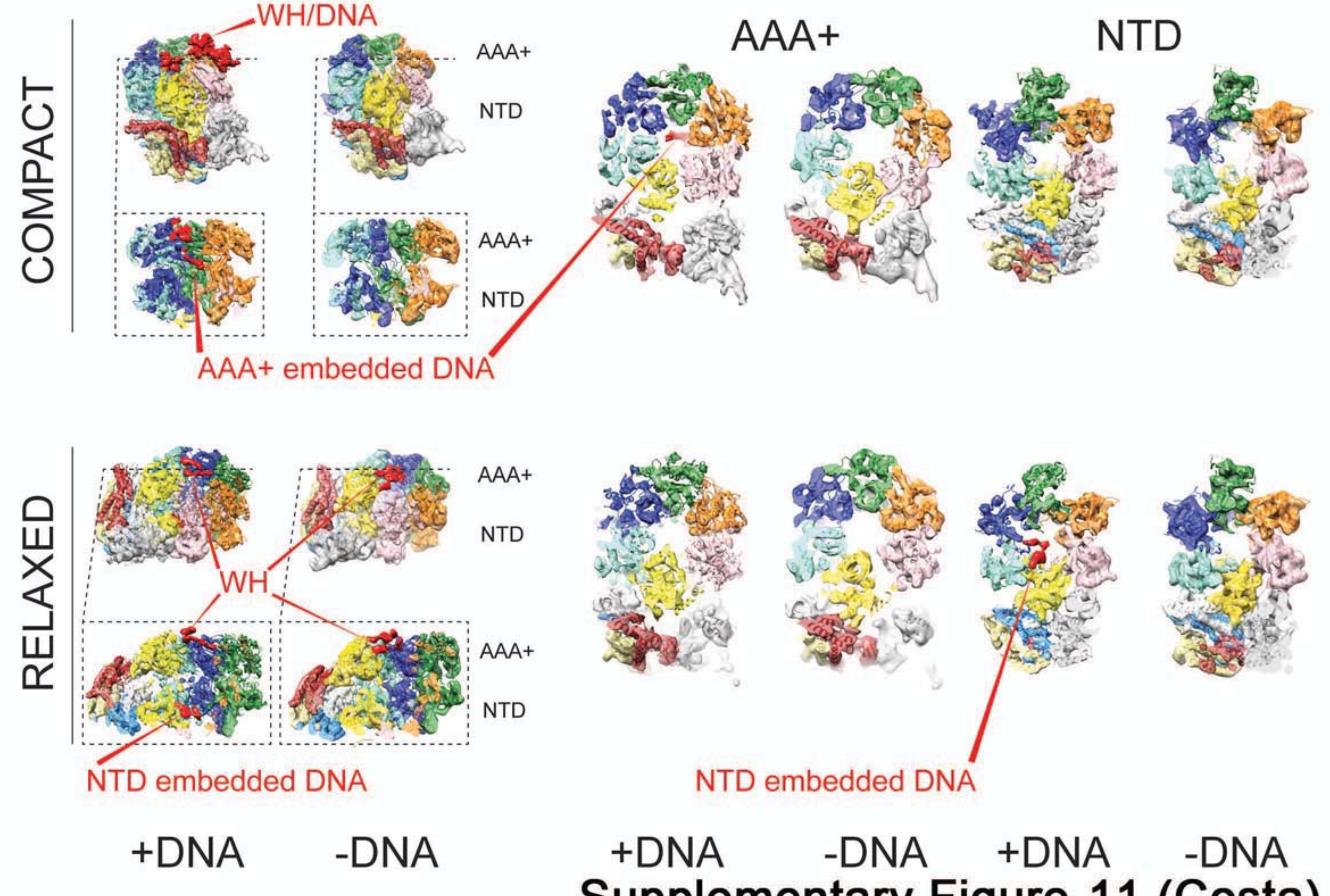

Supplementary Figure-11 (Costa)

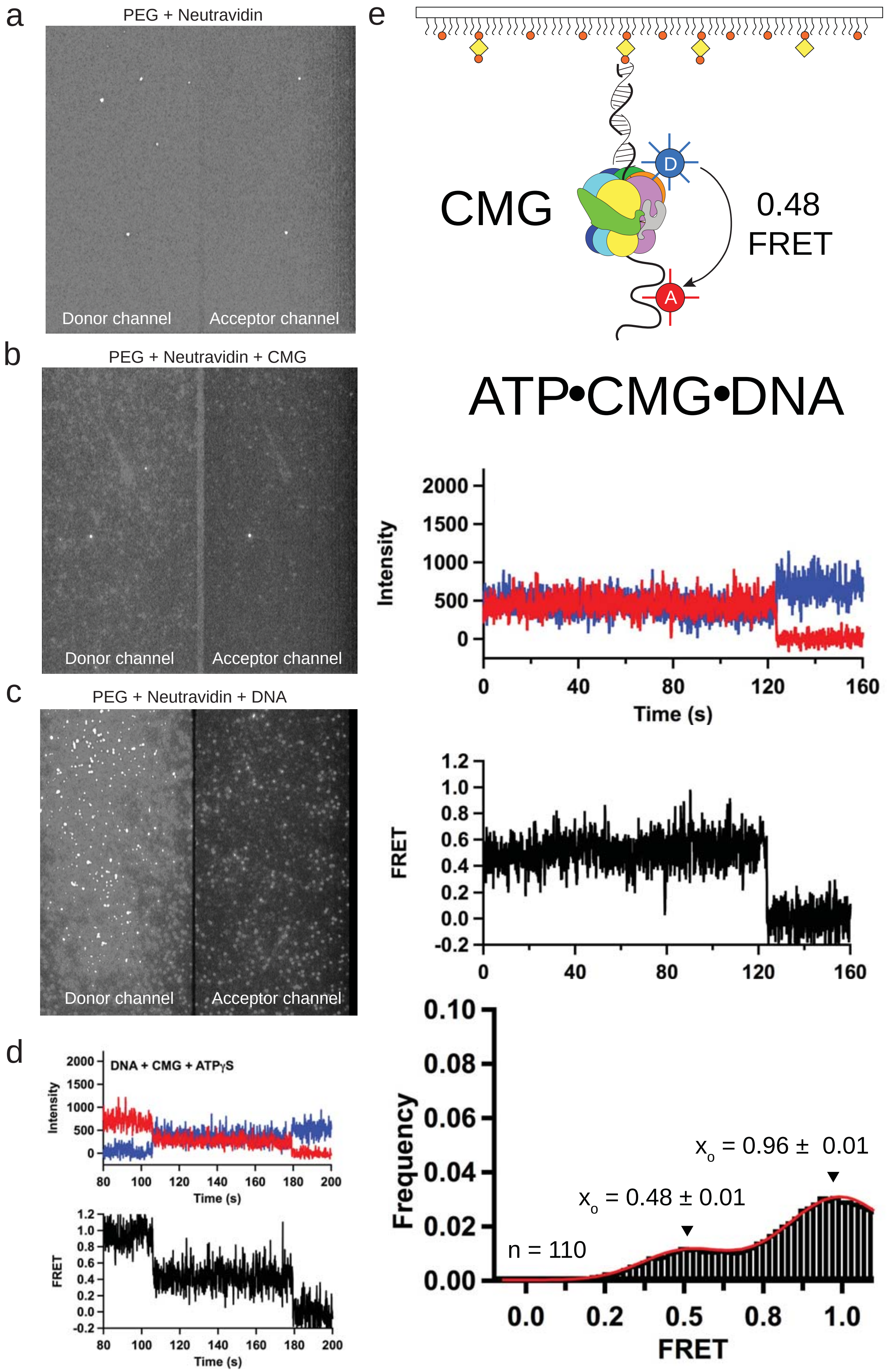

Supplementary Figure 12 (Costa)

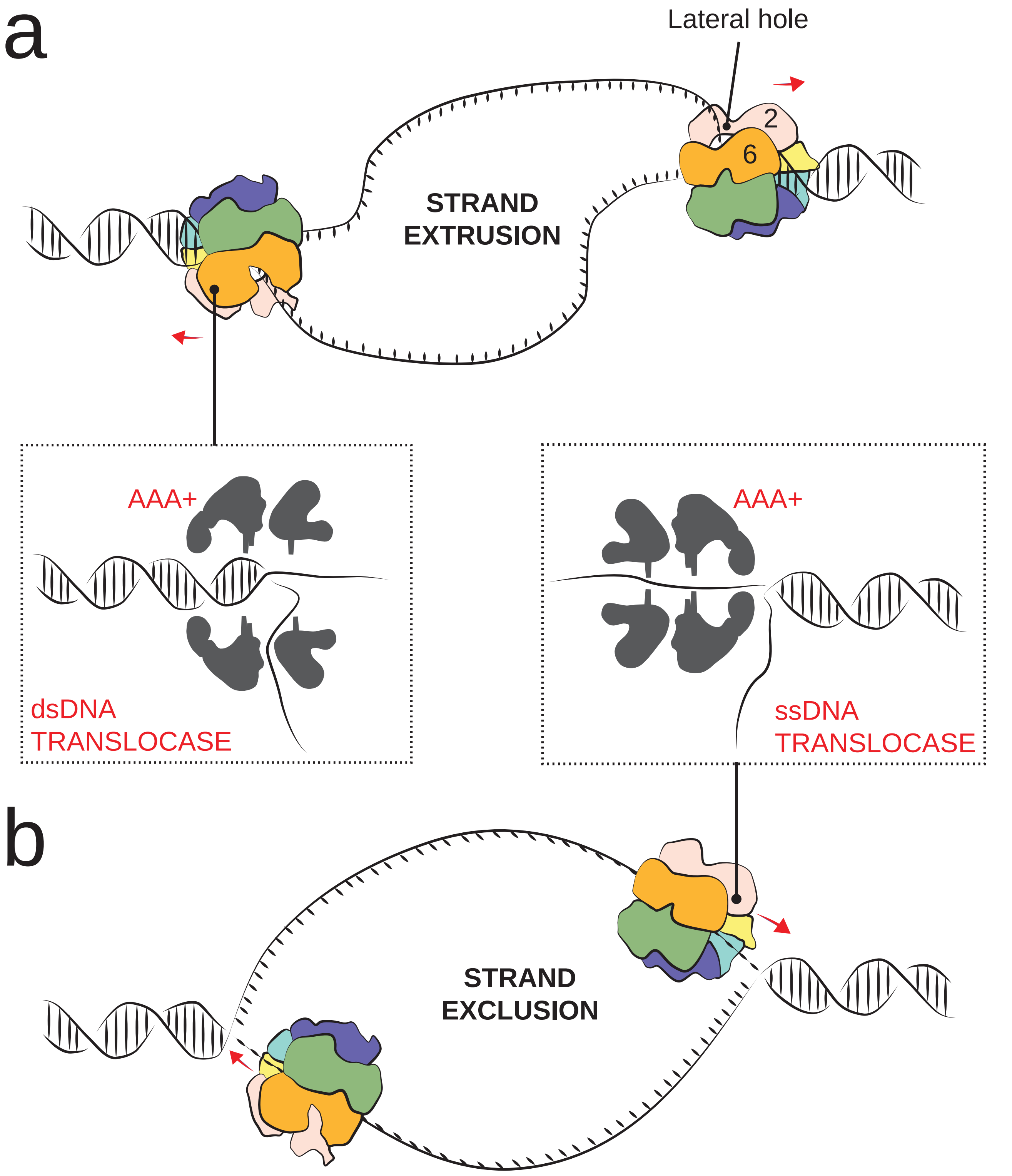

Supplementary Figure 13 (Costa)

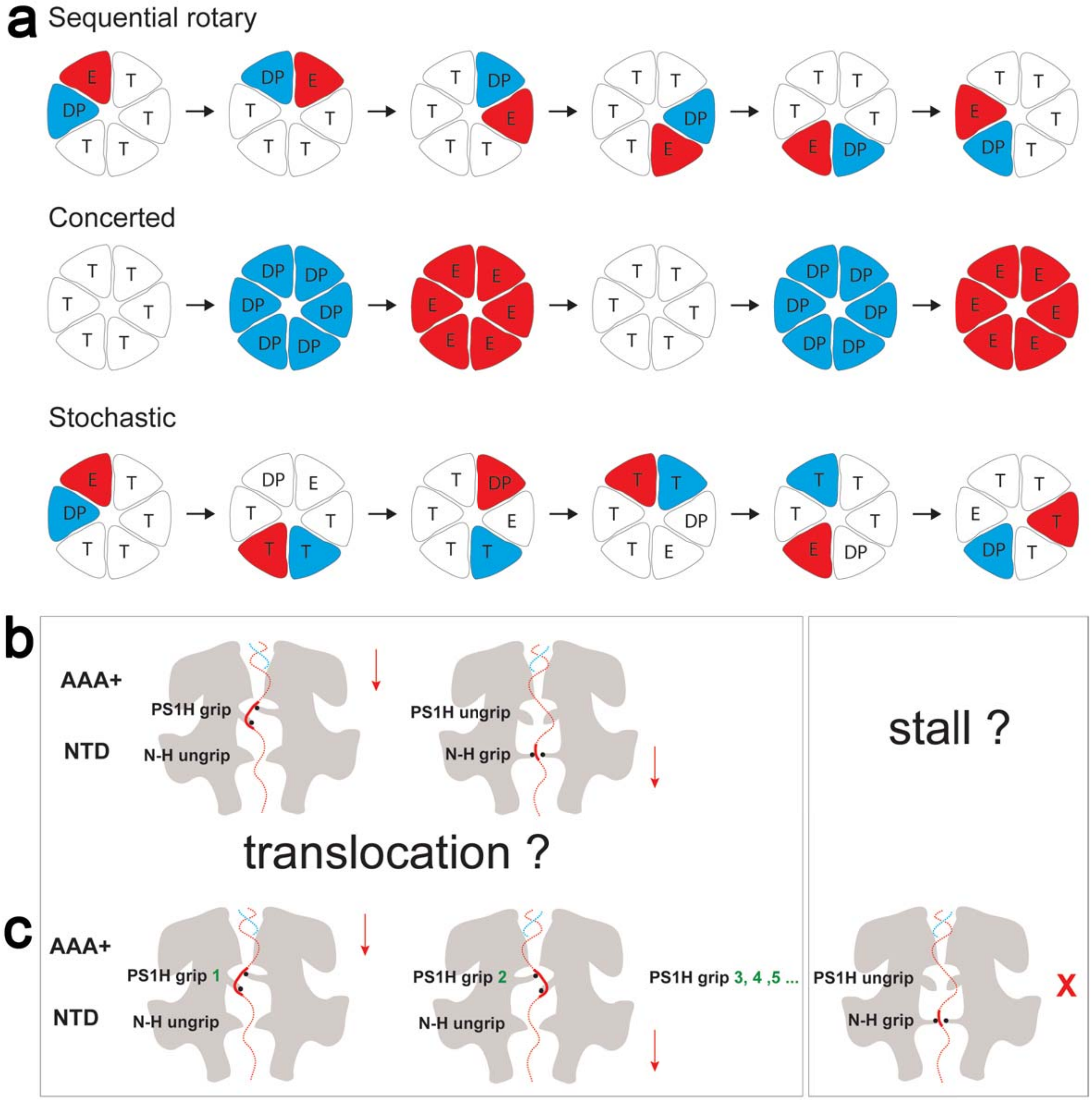

Supplementary Figure-14 (Costa)

Supplementary Figure Legends

Supplementary Fig. 1: Cryo-electron microscopy preparation of the CMG helicase. **(a)** Silver-stained SDS PAGE gel of the purified, 11-member CMG helicase. **(b)** Polyacrylamide gel showing that the CMG helicase is catalytically active. **c)** Cryo-electron micrograph of the ATPγS-CMG-DNA and ATP-CMG preparations. **(d)** Matching between reference free class averages and two-dimensional projection of the ATPγS-CMG-DNA and ATP-CMG 3D volumes (compact ATPase form).

Supplementary Fig. 2: ATPγS-CMG-DNA: Overview of the single-particle reconstruction, 3D classification and structure refinement.

Supplementary Fig. 3: NTD DNA-interacting collar transition between Mcm2-7[4] and the CMG. **(a)** Center of mass rearrangements of the peripheral A domain (blue) and internal B-C domains (yellow) of the N-terminal tier of Mcm2-7. The B-C domains undergo a counter-clockwise, rigid-body transition (when viewed from the C-terminal tier). Arrows represent transition to the CMG. This transition might have a role in helicase activation. The central pore identified by six zinc finger elements (ZFs) constricts as the Mcm2-7 transitions to the active CMG form. **(b)** Cartoon representation of the helicase N-terminal DNA interacting collar in the Mcm2-7[4] (transparent) and CMG (solid).

Supplementary Fig. 4: MCM double hexamer and the CMG. A key dimerization element is represented by an (yeast specific) N-terminal extension of Mcm7 that latches onto the A domain of Mcm5. In the CMG, Psf2 contacts a conserved site on Mcm5 that is protected by the Mcm7 subunit of the opposing hexamer in yeast. We speculate that GINS binding might cause double-hexamer destabilization.

Supplementary Fig. 5: Cdc45 contains a RecJ fold. **(a)** Best docking solution of RecJ (PDB entry 1IR6) into the density assigned to Cdc45 (blue). An alternate solution is shown, with an inverted configuration of the RecJ lobes. **(b)** Fourier-shell correlation between the cryo-EM map and a simulated map derived from the RecJ crystal structure. The FSCs of the best and second best docking solution are compared (7.9 vs 17.7 Å). The best solution closely matches the cryo-EM map resolution (7.4 Å).

Supplementary Fig. 6: Angular distribution and resolution estimation for ATPγS-CMG-DNA (compact ATPase form). **(a)** Euler plot. **(b)** Gold-standard Fourier Shell Correlation. Resolution estimated according to the 0.143 criterion. **(c)** Fourier Shell Correlation comparing the cryo-EM map and atomic model. **(d)** Histogram of resolution distribution. **(e)** Local resolution. **(f)** Surface rendered side view of the ATPγS-CMG-DNA.

Supplementary Fig. 7: Angular distribution and resolution estimation for ATPγS-CMG-DNA (relaxed ATPase form). **(a)** Euler plot. **(b)** Gold-standard Fourier Shell Correlation. Resolution estimated according to the 0.143

criterion. **(c)** Fourier Shell Correlation comparing the cryo-EM map and atomic model. **(d)** Histogram of resolution distribution. **(e)** Local resolution. **(f)** Surface rendered side view of the ATPγS-CMG-DNA.

Supplementary Fig. 8: ATPγS-CMG-DNA: Overview of the single-particle reconstruction, 3D classification and structure refinement.

Supplementary Fig. 9: Angular distribution and resolution estimation for ATP-CMG (relaxed ATPase form). **(a)** Euler plot. **(b)** Gold-standard Fourier Shell Correlation. Resolution estimated according to the 0.143 criterion. **(c)** Fourier Shell Correlation comparing the cryo-EM map and atomic model. **(d)** Histogram of resolution distribution. **(e)** Local resolution. **(f)** Surface rendered side view of the ATP-CMG.

Supplementary Fig. 10: Angular distribution and resolution estimation for ATP-CMG (compact ATPase form). **(a)** Euler plot. **(b)** Gold-standard Fourier Shell Correlation. Resolution estimated according to the 0.143 criterion. **(c)** Fourier Shell Correlation comparing the cryo-EM map and atomic model. **(d)** Histogram of resolution distribution. **(e)** Local resolution. **(f)** Surface rendered side view of the ATP-CMG.

Supplementary Fig. 11: DNA density identification. The DNA-bound and DNA-free forms of the CMG are compared, in both the compact and relaxed ATPase. An apparently bent, rod-like density feature can be recognized in the compact, DNA-CMG structure, which is missing in the DNA-free counterpart.

Although the resolution in this region of the map is limited, the density could easily accommodate duplex DNA and, likely, one or two winged helix appendix mapping on the MCM C-termini. A cut-through view of the MCM reveals an extended density feature (assigned to single-stranded DNA) embedded in the AAA+ domain and contacting Mcm subunits 7, 4 and 6. This feature is missing in the DNA-free counterpart. An end-on view of the AAA+ domain reveals the same AAA+ **/**DNA contact, which cannot be detected in the DNA-free counterpart. The MCM N-terminal domain appears DNA-free. Similar views are shown for the relaxed ATPase form (with or without DNA). Strikingly, the AAA+ face appears DNA free. Some thin density feature surmounting the AAA+ pore is found both in the presence or absence of DNA, and we assign this density to a partially disordered WH domain. End on views reveal a AAA+ domain devoid of any DNA contact and an interaction between single stranded DNA and the NTD collar of MCM. The corresponding DNA-free structures show no unassigned density traversing the central MCM pore.

Supplementary Fig. 12: (a-c) areas of quartz slide imaged with **(a)** PEG+Neutravidin **(b)** PEG+Neutravidin+CMG **(c)** PEG+Neutravidin+DNA showing that FRET signals are only observed when DNA is immobilized to the slide (d) smFRET trajectories for ATPγS CMG in real time where donor (blue) and acceptor (red) intensity trajectories convert to anti-correlated upon CMG binding, until single-step photobleaching of the acceptor occurs (e) cartoon scheme for the smFRET experiment for ATP+CMG+DNA, here DNA is labeled with Cy3 and Cy5 (7 nt separation) attached via a biotin-neutravidin interaction at the slide surface; donor (blue) and acceptor (red) intensity

trajectories are anti-correlated until single-step photobleaching of the acceptor occurs; FRET trajectories (black) show a sharp drop to zero FRET upon acceptor photobleaching; histogram of FRET values collected from all molecules with a Gaussian fit are shown in red, where x_0 is the mean FRET and σ is the distribution width.

Supplementary Fig. 13: Models for DNA fork engagement by the CMG helicase. **(a)** Strand extrusion. The AAA+ motor translocates on duplex DNA. For unwinding, the leading-strand template (translocation strand) exits through the N-terminal collar, while the lagging strand exists through a side opening (possibly at the Mcm2-6 interface). **(b)** Strand exclusion. The MCM is a single-stranded DNA translocase. Only the leading-strand template threads through the MCM toroid, while the lagging-strand template is sterically excluded.

Supplementary Fig. 14: Models for ATP hydrolysis by hexameric ring translocases. ATPase states label: T (white) – ATP state, DP (blue) – ADP + P_i, E (red) – nucleotide exchange. **(a)** Sequential rotary mechanism: subunits bind, hydrolyse and release ATP in an ordered fashion around the ring. Concerted mechanism: all subunits bind, hydrolyse and release ATP at the same time. Stochastic mechanism: ATP binding, hydrolysis and release does not follow a particular order. **(b)** DNA translocation by the MCM motor might involve the hand-off of single-stranded DNA from the AAA+ ATPase domain (PS1 hairpin gripping DNA) to the NTD (N-terminal hairpin, N-H, gripping DNA). **(c)** DNA translocation might involve the rotary cycling of PS1 hairpins

that spool DNA through the ring and the N-H gripping mode might represent a stalled configuration of the helicase.
